# Tetraspanin-enriched microdomains play an important role in pathogenesis in the protozoan parasite *Entamoeba histolytica*

**DOI:** 10.1371/journal.ppat.1012151

**Published:** 2024-10-03

**Authors:** Han Jiang, Herbert J. Santos, Tomoyoshi Nozaki

**Affiliations:** Department of Biomedical Chemistry, Graduate School of Medicine, The University of Tokyo, Hongo, Bunkyo-ku, Tokyo, Japan; Jr., University of Virginia, UNITED STATES OF AMERICA

## Abstract

Tetraspanins (TSPANs) are a family of highly conserved proteins present in a wide variety of eukaryotes. Although protein-protein interactions of TSPANs have been well established in eukaryotes including parasitic protists, the role they play in parasitism and pathogenesis remains largely unknown. In this study, we characterized three representative members of TSPANs, TSPAN4, TSPAN12, and TSPAN13 from the human intestinal protozoan *Entamoeba histolytica*. Co-immunoprecipitation assays demonstrated that TSPAN4, TSPAN12 and TSPAN13 are reciprocally pulled down together with several other TSPAN-interacting proteins including TSPAN binding protein of 55kDa (TBP55) and interaptin. Blue native-PAGE analysis showed that these TSPANs form several complexes of 120–250 kDa. Repression of *tspan12* and *tspan13* gene expression led to decreased secretion of cysteine proteases, while repression of *tspan4* led to a four-fold increase in the activity of cysteine proteases in crude extracellular vesicles (EVs) fraction. Meanwhile, strains overexpressing HA-tagged TSPAN12 and TSPAN13 demonstrated reduced adhesion to collagen. Altogether, this study reveals that the TSPANs, especially TSPAN12 and TSPAN13, are engaged with complex protein-protein interactions and are involved in the pathogenicity-related biological functions such as protease secretion and adhesion, offering insights into the potential regulatory mechanisms of tetraspanins in protozoan parasites.

## Author summary

Tetraspanins (TSPANs) are involved in critical roles such as cellular communication, adhesion, and immune responses. However, their function in parasitic protozoa, particularly in relation to disease-causing mechanisms, remains poorly understood. Our study explores the role of three tetraspanins (TSPAN4, TSPAN12, and TSPAN13) in *Entamoeba histolytica*, an intestinal parasite which causes amebiasis, and a significant health problem affecting millions of people worldwide. Our findings reveal that these amebic TSPANs form various protein complexes similar to what is observed in other organisms. We also found that amebic TSPANs influence crucial processes related to the parasite’s pathogenicity, such as adhesion to collagen and secretion of cysteine proteases (CPs). Gene silencing of TSPAN12 and TSPAN13 caused reduction in CP secretion, while overexpression resulted in decreased adhesion. Additionally, TSPAN4 appears to play a distinct role in exosome-mediated pathways. Our findings provide insights into a potential alternative mechanism of CP secretion via exosomes, in which amebic TSPANs may play a role.

## Introduction

Tetraspanins (TSPANs) are proteins composed of four transmembrane domains (TMDs), a small and a large extracellular loop (SEL and LEL, respectively), and are present in a wide variety of eukaryotes. LEL contains highly conserved characteristic motifs such as cysteine-cysteine-glycine (CCG) motifs. TSPANs are involved in the coordination of intracellular and intercellular functions as signal transduction, pattern recognition, antigen presentation, T-cell proliferation, cell migration, membrane protein trafficking, and membrane compartmentalization [1–5]. Moreover, TSPANs were shown to be involved in infection by several pathogens such as the human immunodeficiency virus (HIV), hepatitis C virus (HCV), human papillomavirus (HPV), *Plasmodium spp*., and *Mycobacterium tuberculosis* [6]. In helminths such as *Opisthorchis viverrine* [7] and *Schistosoma mansoni*, TSPANs play a notable role in the formation of teguments [8], making them potential targets for vaccine development [9]. Furthermore, in the unicellular parasite *Trichomonas vaginalis*, TSPAN TvTsp1 has been identified as a component of exosomes [10], while TvTsp6 was found in the flagella, and was implicated in sensory reception which aids in the parasite’s migration during infection [11].

TSPAN-enriched microdomains (TEMs) in cell membranes, also known as TSPAN clusters, are involved in organizing cellular processes like adhesion and signaling [12]. It has been shown in humans that TSPANs in TEMs bind to a diverse range of molecules, including integrins, lipids, and other TSPANs, and the interaction with integrins has been proven to be pivotal in the regulation of cell adhesion and migration, which are essential processes in immune response and wound healing [13,14]. Additionally, the ability of TSPANs to bind to other TSPANs suggests complex intra-TEM interactions, which are crucial for organizing membrane proteins and modulating signal transduction. Furthermore, their association with lipids points to a role in maintaining membrane integrity and dynamics [15,16]. These microdomains are also crucial in diseases like cancer and immune disorders, mediate critical cell-cell interactions and signaling pathways [17].

Amidst this backdrop, *Entamoeba histolytica* is an intestinal protozoan parasite that causes amebiasis, a neglected cause of morbidity and mortality in low- and medium-income countries. It remains to be a global health problem affecting millions of patients and causing more than 55,000 deaths annually [18]. Disease transmission occurs via ingestion of the infectious cyst through fecally-contaminated food or water. Excystation of cysts after ingestion produces trophozoites which invade the intestinal epithelium [19]. The parasite exploits virulence-related mechanisms which enable the invasion of intestinal epithelial tissue and extraintestinal tissue. Symptoms can vary, ranging from diarrhea, colitis, and dysentery to liver abscess [20].

While the structures and roles of TSPANs in other model eukaryotes including humans are well established [12], they remain largely unknown in *E*. *histolytica*. Our previous in silico genome-wide survey identified a total of 17 TSPANs in *E*. *histolytica* [21], but no functional study nor TEMs composition have been investigated. In this study, we characterized three representative TSPANs in *E*. *histolytica*. We found that these amebic TSPANs are constituents of potential complexes within the TEMs. Our findings prove that *E*. *histolytica* likewise harbors TEMs comprised of a repertoire of distinct, yet consistently present TSPANs and associated binding proteins, analogous to structures observed in other organisms. Our results also indicate multifaceted roles of TSPANs and *Eh*TEMs involving multivesicular bodies (MVBs), exosomes, and the nucleus. Furthermore, we provide evidence linking TSPANs to virulence, notably adhesion and protease secretion, which are hallmark processes in the pathogenesis of *E*. *histolytica*. Specifically, our data indicated a diminished adhesive capability upon independent overexpression of two specific TSPANs. Additionally, targeted gene knock-down of certain TSPANs revealed concomitant changes in cysteine protease secretion. Overall, this study provides new insights into TSPANs and putative binding proteins within *E*. *histolytica*’s TEM hinting at their pivotal roles in the regulation of virulence of this organism.

## Results

### Expression and localization of HA-tagged TSPAN4 in amebic vesicles

**Fig 1:**
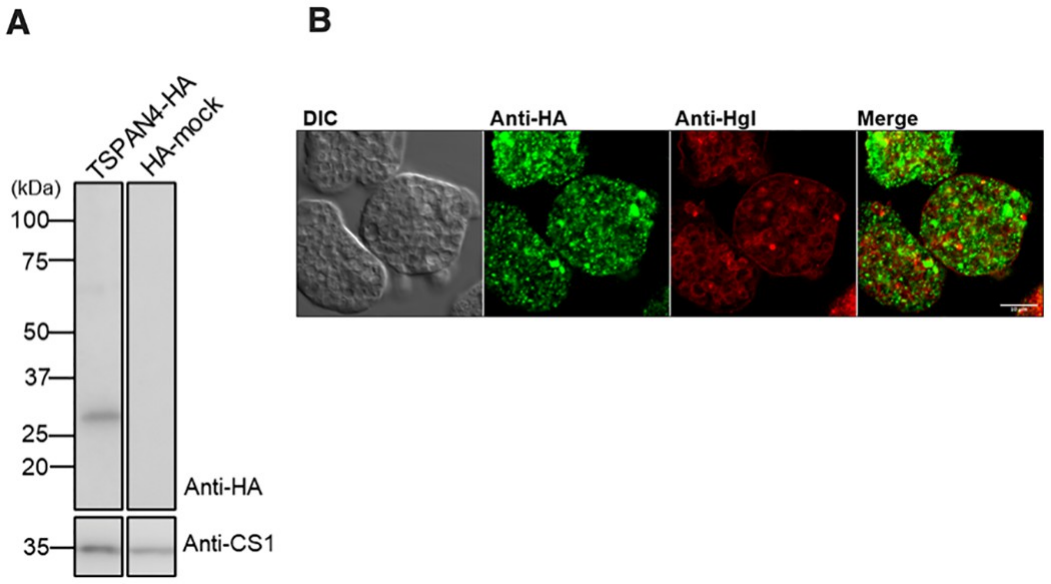
Expression and subcellular localization of TSPAN4-HA. (A) Immunoblot analysis of TSPAN4-HA in *E*. *histolytica* transformants. Approximately 20 μg of lysed cell samples from TSPAN4-HA-expressing transformant (TSPAN4-HA) and HA-mock-expressing transformant (HA-mock) were added to SDS-PAGE and immunoblot analysis using anti-HA antibody. CS1 (cysteine synthase 1) was detected by anti-CS1 antiserum as a loading control. (B) Representative immunofluorescence assay (IFA) micrograph of TSPAN4-HA double-stained with rabbit anti-HA (green) antibody and mouse anti-Hgl (red) antiserum. Scale bar, 10 μm.

Our previous genome-wide search identified 17 TSPANs in *E*. *histolytica* [21]. Among them, TSPAN4 (EHI_075690) was particularly remarkable as the gene was reported to be expressed at a higher level in non-pathogenic strain than its isogenic (i.e., with the identical genetic background) pathogenic strain and that the parasite line that overexpressed TSPAN4 produced smaller amebic liver abscesses in the rodent model [22]. This result indicates that TSPAN4 likely negatively regulates virulence of this parasite in vivo [22]. These observations prompted us to investigate and characterize amebic TSPANs with TSPAN4 being an initial target. To determine the localization of TSPAN4, we created an amebic transformant strain that expresses TSPAN4 with the carboxyl terminus hemagglutinin (HA)-tag (TSPAN4-HA). Immunoblot analysis of TSPAN4-HA overexpressing strain with anti-HA antibody confirmed expression of TSPAN4-HA as a single band corresponding to the predicted size of 27 kDa (Fig 1A). We analyzed the subcellular localization of TSPAN4-HA by immunofluorescence assay (IFA). To assess whether TSPAN4 is localized to the plasma membrane, similar to TSPANs in other organisms, we performed double staining IFA with anti-HA antibody and a plasma membrane marker anti-Hgl [galactose and N-acetyl-galactosamine (Gal/GalNAc) lectin heavy subunit] monoclonal antibody. Our IFA result showed that the primary location of TSPAN4-HA is not the plasma membrane, but internal vesicles (Fig 1B).

### TSPAN4 interacts with other TSPANs and various other partner proteins

**Fig 2:**
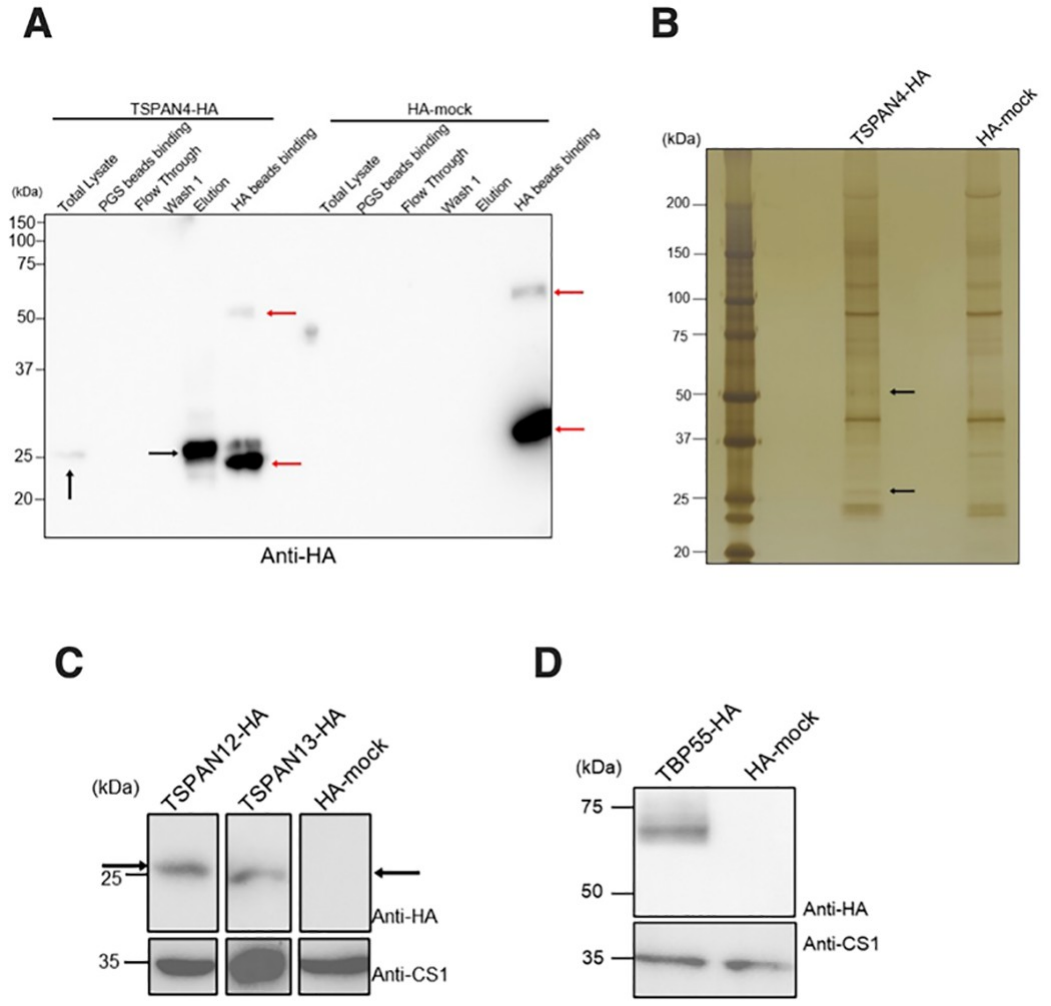
Co-immunoprecipitation assay of TSPAN4-HA. (A) Representative immunoblot analysis and silver staining of TSPAN4-HA in co-IP experiments. 5 μl of each fraction collected from co-IP experiments were added into SDS-PAGE and immunoblot analysis using anti-HA antibody. Black arrows indicate TSPAN4-HA expression and red arrows indicate the heavy and light chain of anti-HA antibody. (B) Silver staining was performed by adding 10 μl of eluted fraction of TSPAN4-HA and HA-mock in the co-IP. The black arrows suggest specific bands in the TSPAN4-HA sample. (C and D) Immunoblot analysis of TSPAN12-HA, TSPAN13-HA and TBP55 in *E*. *histolytica* transformants. Black arrows indicate TSPAN12-HA and TSPAN13-HA respectively.

To better understand the function of TSPAN4, we aimed to identify its interacting proteins by performing co-immunoprecipitation (co-IP) assay on TSPAN4-HA expressing trophozoites. Co-IP validation was performed by western blot analysis using anti-HA antibody. TSPAN4-HA was successfully precipitated and eluted (Fig 2A). The eluted fractions from the TSPAN4-HA and mock-HA co-IP samples were subjected to SDS-PAGE and silver staining (Fig 2B). The silver-stained gel showed unique bands detected in the TSPAN4-HA lane, specifically with an approximate molecular weight of 25 and 50 kDa, which were absent in the control mock-HA lane (Fig 2B, black arrows). The elution samples for both TSPAN4-HA and mock control were analyzed via mass-spectrometry (MS) to identify the binding partners of TSPAN4. Three independent co-IP experiments followed by MS analysis were performed to confirm reproducibility of the data.

To analyze the proteome data from the mass-spectrometry analysis, we prepared two lists of proteins labeled as “exclusive hits” and “enriched hits”. Exclusive hits indicate the protein candidates that were specifically pulled-down by HA-tagged TSPAN4 samples and not in the mock control. For the enriched hits, the ratio of quantitative value (QV) of normalized total spectra between HA-tagged TSPAN4 and mock control was computed and a threshold QV ratio of 2.0 and above was set. A summarized table (S1 Table) was prepared, containing exclusive and enriched hits in all three independent co-IP trials, irrespective of QV, as well as proteins that have a mean QV over 5.0 in two out of three independent co-IP trials. The complete pull-down results are available as S1 File.

A total of four proteins were exclusively pulled down while one protein was enriched in TSPAN4-HA compared to mock-HA in all three trials (S1 Table). Moreover, TSPAN4 was validated to be exclusively precipitated in all three trials. Interestingly, two other TSPANs, TSPAN12 (EHI_091490) and TSPAN13 (EHI_107790) were likewise exclusively detected in all three trials with comparatively high mean QV of 13.6 and 19.0, respectively. This data implies that in *E*. *histolytica* TSPANs forming complexes called tetraspanin-enriched microdomains (TEMs) is also conserved. In addition, two other proteins EHI_001100 and EHI_148910 were also pulled down in all three trials with the mean QV of 105.3 and 6.8, respectively.

### *E*. *histolytica* TSPAN4, TSPAN12, and TSPAN13 form heterogeneous tetraspanin-enriched microdomains (TEMs)

Upon identification of TSPAN12 and TSPAN13 as interacting proteins of TSPAN4-HA, we next constructed respective plasmids to express TSPAN12-HA and TSPAN13-HA in *E*. *histolytica* trophozoites. We confirmed the expression of TSPAN12-HA and TSPAN13-HA by anti-HA immunoblot analysis of corresponding cell lysates at approximately 28 kDa and 25 kDa, respectively (Fig 2C). To prove the potential direct or indirect interactions between these TSPANs, we performed reverse co-IP targeting TSPAN12-HA and TSPAN13-HA, followed by protein sequencing of eluted fractions by mass-spectrometry analysis. The co-IP and MS analyses were likewise independently repeated three times. Co–IP of TSPAN12-HA (S1A Fig) and TSPAN13-HA (S2A Fig) were validated as anti-HA immunoblotting revealed TSPAN12-HA and TSPAN13-HA bands appeared in respective eluted fractions. We also detected specific bands at around 25 and 50 kDa for TSPAN12 and 50 kDa for TSPAN13, which are supposed to be the potential binding partners of TSPAN12 and TSPAN13 in the silver-stained SDS-PAGE gels loaded with eluted fractions from mock control, TSPAN12-HA (S1B Fig) and TSPAN13-HA (S2B Fig). The results in the list for TSPAN12-HA (S2 Table) showed that the interacting proteins had a similar pattern with that of TSPAN4-HA. A total of seven proteins were pulled down by all three TSPAN12-HA co-IP trials. This includes TSPAN4, TSPAN12, TSPAN13, EHI_001100, and EHI_148910, which were also pulled down in all three trials of the TSPAN4-HA co-IP. Of note, EHI_001100 has a high QV compared with other binding partners of TSPAN4 and TSPAN12. This protein has been consistently identified in multiple independent co-IP experiments, indicating it is a potential interacting protein of both TSPAN4 and TSPAN12. Hence, we named it TSPAN-binding protein of 55 kDa (TBP55). Another protein, EHI_148910, was detected with the mean QV of 6.8, 13.0, and 31.1 in all three trials of TSPAN4-HA, TSPAN12-HA and TSPAN13-HA, respectively. This protein was annotated as interaptin in AmoebaDB (https://amoebadb.org/), so we named it *Eh*interaptin.

On the other hand, the interactome of TSPAN13-HA showed a different pattern with that of TSPAN4 and TSPAN12. There are in total 57 proteins pulled down by all three trials of TSPAN13-HA co-IP, and neither TSPAN4 nor TSPAN12 were reversely pulled down by TSPAN13-HA (S3 Table). In addition, TBP55, although also pulled down in all three trials, only had a mean QV of 2.4 as compared to 105.3 and 65.5 in the TSPAN4-HA and TSPAN12-HA proteomic data, respectively. The complete pull-down results are available as S1 File.

### TBP55 is a key binding partner of amebic TSPANs

To further investigate the non-TSPAN binding partners of TSPAN4-HA and TSPAN12-HA, we established a TBP55-HA strain. Expression of TBP55-HA was successfully confirmed in the total lysate of *E*. *histolytica* (Fig 2D) by anti-HA immunoblotting. Co-IP of TBP55-HA was also performed three times (S3 Fig). The following MS analysis of TBP55-HA co-IP samples showed consistent pull down of TSPAN4, TSPAN12 and TSPAN13 in *E*. *histolytica* (Table 1). TBP55 also pulled down EHI_165070 at the mean QV of 17.1 in all three trials. EHI_165070 is annotated as short chain dehydrogenase, and was identified to be involved in fatty acid elongation [23]. Such binding suggests that TBP55 may associate with fatty acid elongation. Another enzyme EHI_076870, annotated as steroid 5-alpha reductase, was hit by TBP55-HA co-IP twice with a mean QV of 54.9. Steroid 5-alpha reductase was associated with amebic cell motility [24]. EHI_164540, annotated as β-ketoacyl reductase, was also detected twice in the TBP55-HA co-IP proteome. These three membrane proteins are supposed to locate to the ER membrane, which may also be one localization of TBP55. Besides, G protein-coupled receptor 1 (EhGPCR1, EHI_025100) was detected twice in the proteome with a mean QV of 8.0. GPCRs are considered as conserved interacting partners of TEMs [25].

**Table 1:** Mass-spectrometry results of HA-tagged TBP55 co-immunoprecipitation. Co-IP assay followed by mass-spectrometry analysis were performed as described in Materials and methods. Frequency of identification indicates the frequency for one protein to be detected in an exclusive or enriched manner in three independent trials. Mean of quantification value suggests the mean of quantitative value (normalized total spectra) calculated by Scaffold 5 software, the value outside the parenthesis stands for HA-tagged TBP55 sample while the value inside the parenthesis stands for mock control. The order is sorted by frequency of identification firstly, and the mean of quantitative value secondly.

Accession number	Frequency of identification	Mean of quantitative value	Annotation
EHI_001100	3	267.8 (0.9)	TBP55
EHI_107790	3	62.8 (0)	TSPAN13
EHI_091490	3	17.7 (0)	TSPAN12
EHI_165070	3	17.1 (4.3)	Short chain dehydrogenase
EHI_076870	2	54.9 (20.0)	Steroid 5-alpha reductase
EHI_040700	2	24.0 (9.1)	Coatomer subunit gamma
EHI_054830	2	19.3 (6.0)	Calcium-transporting ATPase
EHI_163540	2	14.3 (2.9)	β-ketoacyl reductase
EHI_020300	2	13.6 (5.7)	Ribosomal protein L15
EHI_075690	2	12.5 (0)	TSPAN4
EHI_163240	2	9.7 (3.4)	Phosphatidate cytidylyltransferase
EHI_179320	2	9.1 (3.3)	Wntless-like transmembrane protein
EHI_011940	2	8.4 (2.2)	Dolichyl diphosphooligosaccharide protein glycotransferase
EHI_025100	2	8.0 (2.0)	G protein-coupled receptor, EhGPCR1
EHI_096210	2	6.6 (2.2)	Coated vesicle membrane protein
EHI_042870	2	6.4 (0)	Cell surface protease gp63, putative
EHI_086380	2	6.2 (2.5)	Hypothetical protein
EHI_068200	2	5.5 (2.5)	60S ribosomal protein L31, putative
EHI_044830	2	5.2 (2.3)	60S ribosomal protein L39, putative
EHI_093790	2	5.0 (0)	Dolichyl-diphosphooligosaccharide—protein glycosyltransferase subunit 1

### Nuclear proteins were pulled down with TSPANs and *Eh*interaptin

We also tried co-IP experiments for *Eh*interaptin-HA and the mock control strains. The validation western blot showed band for the correct size of *Eh*interaptin-HA (S4 Fig). The results of mass spectrometry analysis identified multiple nuclear proteins that were pulled down by *Eh*interaptin (S4 Table). Based on the following and previous mass spectrometry analysis (S1–S4 Tables) several nuclear pore complex (NPC) proteins were identified in the pull-down assays of TSPANs and *Eh*interaptin. Nucleoporin210 (Nup210, EHI_183510) was bound by TSPAN4-HA with a comparatively high mean QV of 22.3 in two out of three trials (Table 2). It was also pulled down by TSPAN12-HA with a QV of 7.6 in one out of three trials (Table 2). Moreover, TSPAN13-HA also pulled down Nup210 with a high mean QV of 29.0 in all three trials (Table 2). Another NPC protein Nucleoporin 54 (Nup54, EHI_010010) was pulled down by TSPAN12-HA with a QV of 6.8 in one out of three trials (Table 2), and by TSPAN13-HA with a mean QV of 9.5 in all three trials (Table 2). The nuclear pore complex serves as a critical gateway for the transport of molecules between the nucleus and the cytoplasm, regulating the passage of proteins, RNA, and other macromolecules to maintain cellular functions [26]. Nup210 and Nup54 are key components of this intricate structure, playing distinct roles in mediating nucleocytoplasmic transport and contributing to the overall functionality of the nuclear pore complex [27,28]. Overall, the pulldown of various nuclear proteins in TSPANs and the robust binding between TSPANs and *Eh*interaptin indicate the potential nuclear functions for TSPANs, especially TSPAN13. *Eh*interaptin also pulled down many nuclear proteins such as nucleolar protein 56 (Nop56, EHI_122740) with mean QV of 17.7 in all two trials. Nup210 again was pulled down by *Eh*interaptin the but only in one trial with a QV of 18.7, centromere binding protein cbf5 (EHI_115300) with a mean QV of 6.8 in all two trials and several ribosomal proteins including 40S ribosomal protein S6 (EHI_000590) with QV of 18.9 and 60S ribosomal protein L16-B (EHI_177190) with QV of 5.2. Uniquely, when looking at the list of binding proteins of TSPAN13-HA, there are more nuclear proteins than the other two TSPANs and TBP55 (S3 Table). For instance, there are Nucleoporin 210 (Nup210, EHI_183510), two SMC domain-containing proteins (*Eh*interaptin, EHI_152940), Cell division cycle protein 48 (EHI_045120), and Nucleoporin 54 (Nup54, EHI_010010). Several nuclear pore complex (NPC) members identified in the pull-down using TSPAN13-HA as bait indicate that TSPAN13 may be involved directly or indirectly in nuclear transport. Using cNLS Mapper prediction, a tool that identifies importin α-dependent nuclear localization signals (NLSs), https://nls-mapper.iab.keio.ac.jp), TSPAN13 possesses a bipartite NLS signal from positions 107 to 140.

**Table 2:** Nuclear proteins pulled-down by amebic TSPANs and their binding proteins. Nucleus-localized proteins found in TSPANs and *Eh*interaptin’s respective co-IP proteomes. The values on the upper cell of TSPAN4-HA, TSPAN12-HA, TSPAN13-HA, *Eh*interaptin-HA indicate the number of independent trials the protein was pulled down by the respective bait. The values on the lower cell indicate mean QV; number outside parenthesis is QV in TSPANs or *Eh*interaptin, while number inside parenthesis is QV in the mock control. ND, no detection. QV represents quantitative value, normalized with total spectral counts.

Accession number	Annotation	TSPAN4-HA	TSPAN12-HA	TSPAN13-HA	*Eh*interaptin-HA
EHI_183510	Nup210	2 out of 3	1 out of 3	3 out of 3	1 out of 2
		22.3 (0)	7.6 (0)	29.0 (1.9)	18.7 (3.2)
EHI_010010	Nup54	ND	1 out of 3	3 out of 3	1 out of 2
			6.8 (0)	9.5 (3.5)	4.9 (1.3)
EHI_122740	Nop56	1 out of 3	ND	ND	1 out of 2
		10.1 (4.8)			17.7 (8.2)
EHI_115300	Cbf5	ND	1 out of 3	ND	2 out of 2
			14.1 (4.0)		6.8 (1.9)
EHI_148910	*Eh*interaptin	3 out of 3	2 out of 3	3 out of 3	2 out of 2
		6.8 (0)	13.0 (1.1)	31.1 (1.5)	85.5 (1.9)
EHI_152940	SMC-domain containing protein	2 out of 2	2 out of 3	3 out of 3	1 out of 2
		8.0 (0)	4.2 (3.8)	15.1 (1.5)	56.2 (0)

### Cellular localization of TSPAN12, TSPAN13, TBP55, and *Eh*interaptin

**Fig 3:**
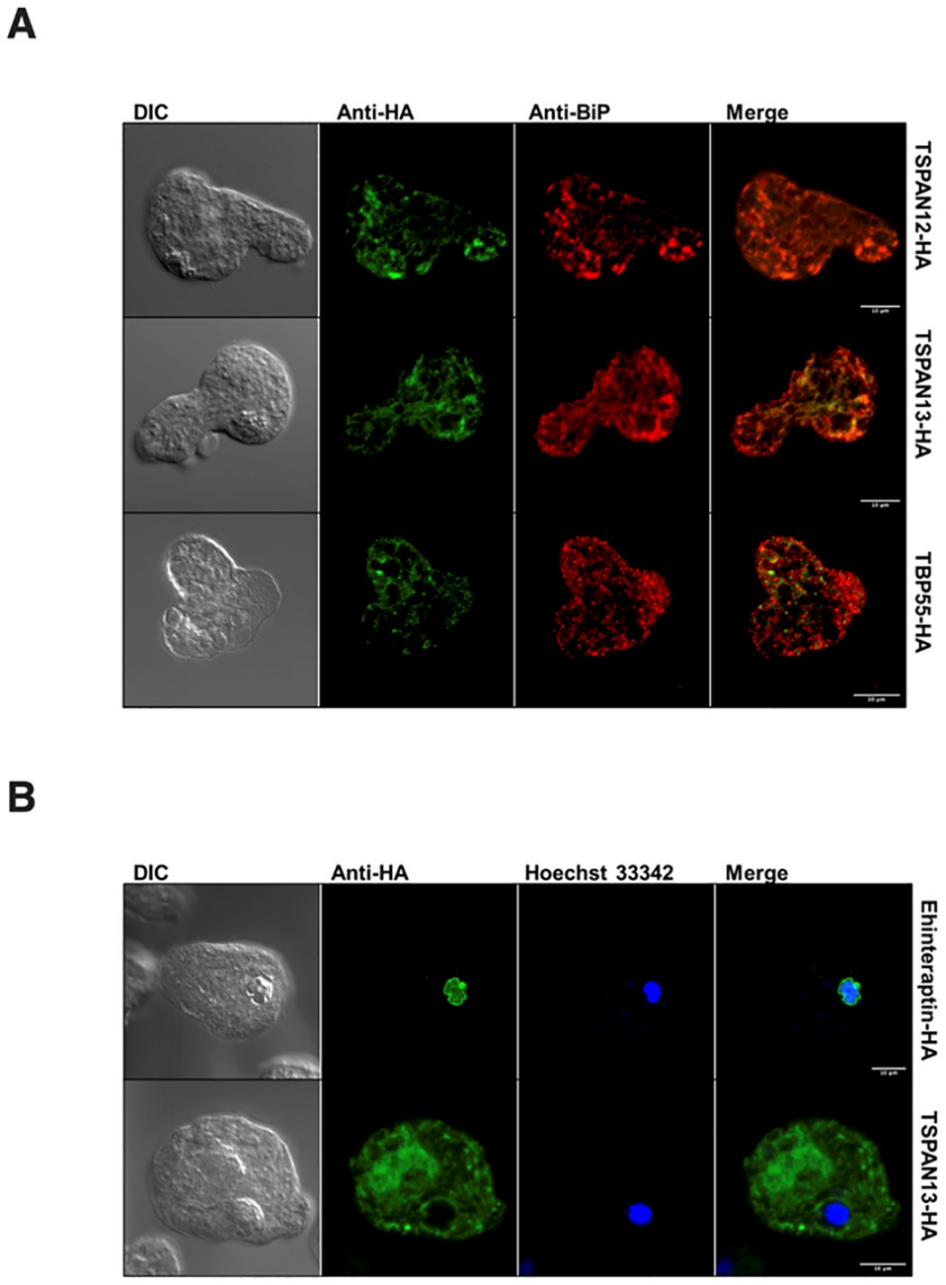
Subcellular localization of TSPAN12-HA, TSPAN13-HA, TBP55-HA and *Eh*interaptin-HA. (A) Representative immunofluorescence assay micrographs of TSPAN12-HA (top panel), TSPAN13-HA (middle panel) and TBP55-HA (bottom panel) double-stained with mouse anti-HA (green) antibody and rabbit anti-BiP (red) antiserum respectively. Scale bar, 10 μm. (B) Representative immunofluorescence assay micrographs of *Eh*interaptin-HA and TSPAN13-HA double-stained with mouse anti-HA antibody (green) and DNA binding dye Hoechst 33342 (blue) respectively. Scale bar, 10 μm.

Although the subcellular localization of three TSPANs (TSPAN1, 2 and 4) in *E*. *histolytica* was described as mostly cytosolic vesicular compartments in our previous [21] and present studies, the subcellular localization of other TSPANs remains unknown. To further clarify the subcellular localization of TSPAN12, TSPAN13, and their binding partners, TBP55 and *Eh*interaptin, the IFAs were performed on the transformants that expressed TSPAN12-HA, TSPAN13-HA, TBP55-HA, and *Eh*interaptin-HA, respectively. The anti-HA signals of fixed TSPAN12-HA, TSPAN13-HA and TBP55-HA trophozoites were similar to the punctate patterns also observed in our TSPAN4-HA IFA (Fig 3A). Double-staining IFA using anti-HA antibody and anti-binding immunoglobulin protein (BiP) antiserum (ER marker, control) were used to check the subcellular localization of TSPAN12-HA, TSPAN13-HA and TBP55-HA. We found that TSPAN12-HA, TSPAN13-HA and TBP55-HA show partial colocalization with the ER marker (anti-BiP) having colocalization coefficients of 0.652, 0.569 and 0.226, respectively (Fig 3A). Representative scatter plots used in evaluating colocalization are provided (S5 Fig). Meanwhile, *Eh*interaptin-HA showed obvious nuclear localization by Hoechst 33342 nuclear staining (Fig 3B, top panel). Additionally, TSPAN13-HA appears to be localized in close proximity to the nucleus, forming a distinct ring-like structure that encircles the immediate perinuclear region, as observed in the DIC image (Fig 3B, bottom panel).

### Blue native-PAGE revealed heterogeneous complexes of TSPAN4-HA, TSPAN12-HA, and TBP55-HA

**Fig 4:**
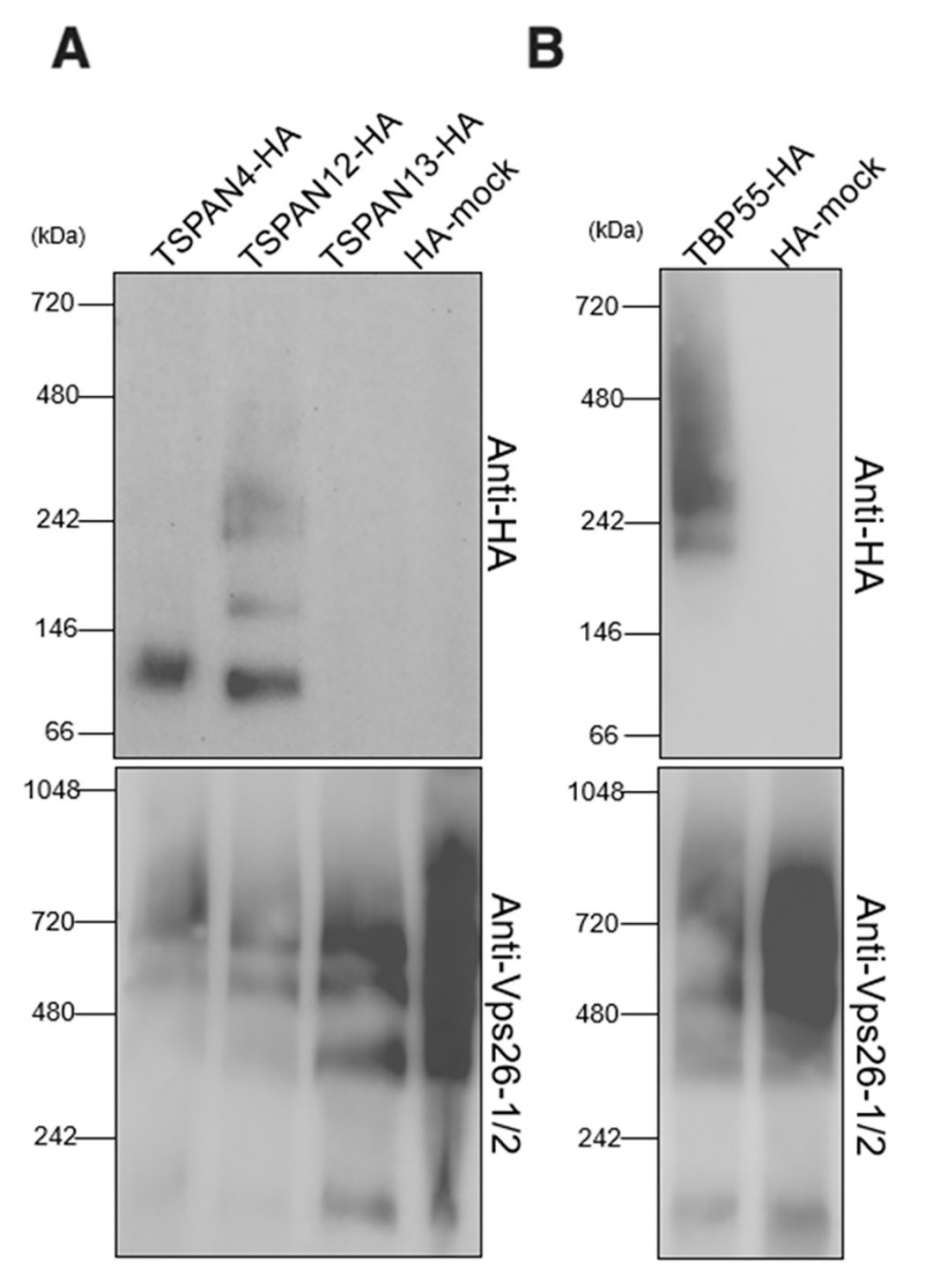
Blue native-PAGE indicates TSPAN4-HA, TSPAN12-HA and TBP55-HA form complexes in digitonin-solubilized organelle-enriched fraction. Transformants expressing (A) TSPAN4-HA, TSPAN12-HA, TSPAN13-HA and (B) TBP55-HA together with HA-mock control were used for subcellular fractionation and BN-PAGE. Immunoblot analyses were performed by anti-HA antibody, and anti-Vps26-1/2 antiserum as loading control, respectively.

To investigate potential complex formation and determine the approximate size of the individual TSPAN-associated complexes at TEMs comprising of TSPAN4-HA, TSPAN12-HA, TSPAN13-HA, or TBP55-HA, we conducted organelle membrane fractionation followed by blue native (BN)-PAGE using the transformants of TSPAN4-HA, TSPAN12-HA, TSPAN13-HA, TBP55-HA, and mock-HA strains. Protein complexes were found in the digitonin-solubilized organelle-enriched fraction of trophozoites expressing TSPAN4-HA, TSPAN12-HA, TBP55-HA, but not TSPAN13-HA. A complex of approximately 100 kDa containing TSPAN4-HA was identified based on immunoblot analysis of the BN-PAGE separated organelle-enriched fraction (Fig 4A). On the other hand, four complex bands were detected after anti-HA immunoblotting of the BN-PAGE separated organelle-enriched fraction of TSPAN12-HA at around 250, 230, 160, and 100 kDa (Fig 4A). Meanwhile, TBP55-HA was determined to form two complexes with approximate molecular weights of 250 and 230 kDa (Fig 4B). Anti-Vps26-1/2 was utilized as a loading control [29] targeting amebic Vps26, a component of the retromer complex which is involved in the retrograde transport of hydrolase receptors from the endosomes to the trans-Golgi [30].

### TSPANs are involved in both adhesion and cysteine protease secretion in *E*. *histolytica*

**Fig 5:**
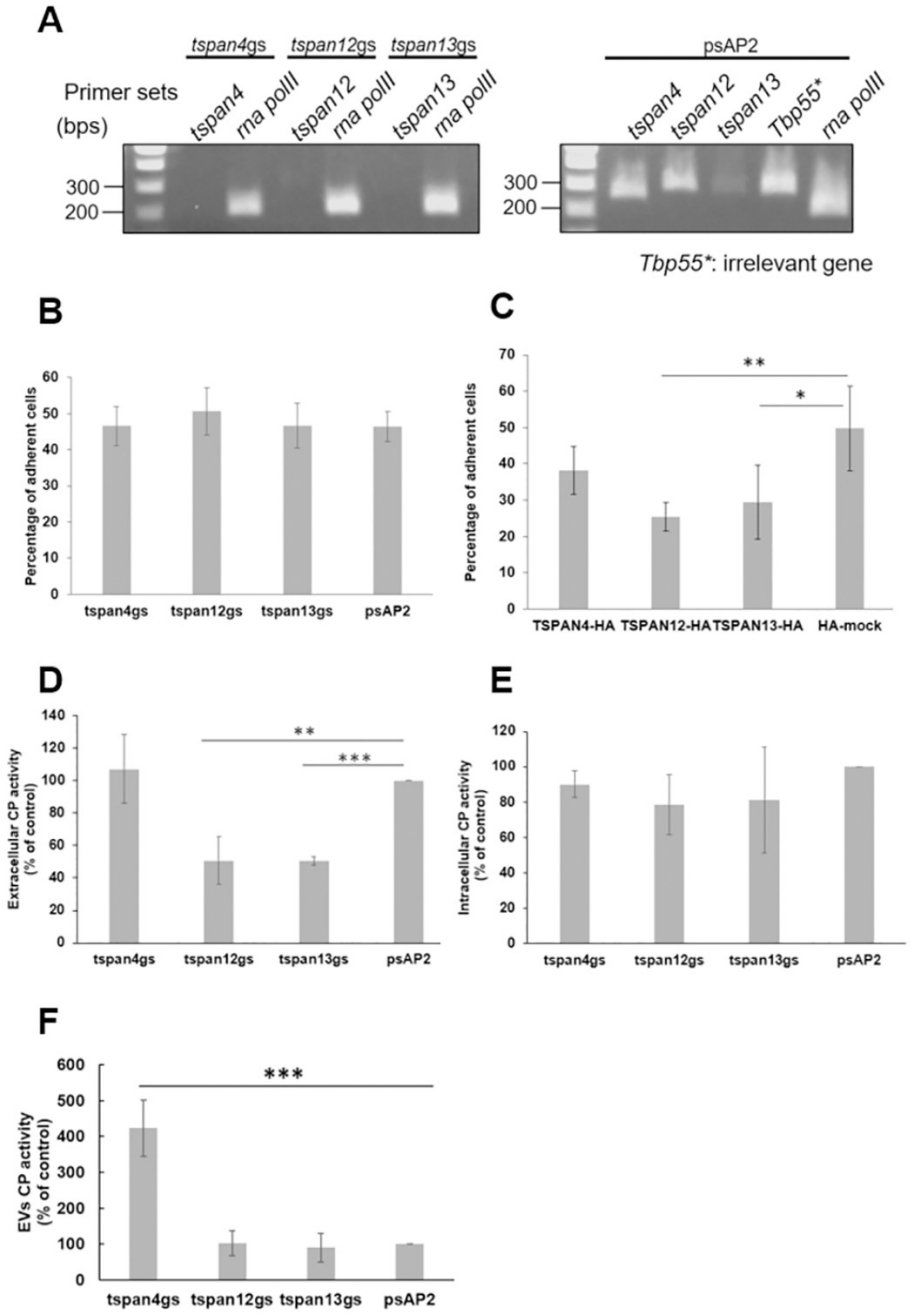
Tetraspanins variably affect the virulence of *E*. *histolytica*. (A) Confirmation of gene silencing by RT-PCR analysis of mock transfected and psAP2 derived gene silenced (GS) strains. Transcripts of *tspan4*, *tspan12*, *tspan13*, *tbp55* and *rna polymerase II* genes were amplified by RT-PCR from cDNA isolated from the transformants and examined by agarose gel electrophoresis. (B) Adhesion of CellTracker Green-stained *tspan4*, *tspan12* and *tspan13* gene silenced strains and control. Fluorescence intensity was detected before and after washing. The assay was performed five times independently. Data points in the graph show the mean and error bars represent standard deviation for five trials. Statistical significance was examined with Student’s unpaired t-test. (C) Transformants expressing TSPAN12-HA and TSPAN13-HA showed lower adhesive ability against HA-mock control strain. (** P < 0.01, p-value = 0.0078; * P<0.05, p-value = 0.0395). (D) *tspan*12 and *tspan*13 gene silenced strains have comparatively lower CP activity in extracellular environment, (E) While there is no significant difference in intracellular CP activity. Intracellular CP activity measurement was based on cell lysate and extracellular CP activity measurement was based on the supernatant after two centrifugations of 3,000 g and 15,000 *g* respectively. Samples were mixed with substrate z-Arg-Arg-MCA and underwent 60 minutes of fluorescence intensity detection. Data points in the graph show the mean and standard deviation for a total three independent trials with triplicates samples. Statistical significance was examined with Student’s unpaired t-test (*** P < 0.001, p-value = 0.0000043, ** P<0.01, p-value = 0.0042). (F) CP activity in crude EV fractions of *tspan4*gs is four-fold higher than the control. Permeabilized crude EV samples were mixed with substrate z-Arg-Arg-MCA and underwent 60 minutes of fluorescence intensity detection. Data points in the graph show the mean and standard deviation for a total four independent trials with triplicates samples. Statistical significance was examined with Student’s unpaired t-test (*** P<0.01, p-value = 0.0007). Readout data for Fig 5B to F are available in S1 Dataset.

Regulation of cell adhesion is considered as one feature of TSPAN family proteins [31]. Adhesion to collagen-coated plates, mimics the intestinal extracellular matrix, which the parasite must adhere to during infection to allow for tissue invasion, phagocytosis/trogocytosis, and cytotoxicity. Thus, an in-vitro adhesion assay on collagen-coated plastic plate was performed on amebic lines in which each of *tspan4*, *tspan12*, and *tspan13* genes were individually silenced. To this end, *E*. *histolytica* G3 strains transformed with psAP2-TSPAN4, psAP2-TSPAN12, psAP2-TSPAN13, and psAP2 (control) were used for gene silencing strain establishment [32]. The respective genes for *tspan4*, *tspan12*, *tspan13* were almost completely abolished based on the electrophoresis of PCR-amplification of corresponding transcripts (Fig 5A). The mean adhesive cell percentage of *tspan4*gs, *tspan12*gs, *tspan13*gs and psAP2 control transformants was 46.6% ± 5.3%, 50.6% ± 6.5%, 46.6% ± 6.1%, and 46.4% ± 4.2%, respectively (Fig 5B). The P-values of unpaired t-test of all the gs strains are over 0.05, suggesting that *tspan* gene silencing did not affect adherent ability with statistical significance.

We also examined adhesive ability of transformants expressing TSPAN4-HA, TSPAN12-HA, TSPAN13-HA, and HA-mock. The experiments were repeated for four independent trials. The mean adhesive cell percentage of TSPAN4-HA, TSPAN12-HA, TSPAN13-HA, and HA-mock were measured at 38.2% ± 6.6%, 24.5% ± 4.0%, 29.5% ± 10.1% and 49.8% ± 11.7%, respectively (Fig 5C). The results suggested that TSPAN12-HA and TSPAN13-HA strains showed statistically significant lower adhesion ability (*p* = 0.0078, 0.0395, respectively) than the HA-mock control strain. Notably, TSPAN12-HA overexpressing trophozoites showed almost half the number of adhesive cells compared to HA-mock control. The robustness of the cell adhesion assay was validated by the linear correlation between cell dye stained trophozoites cell number and fluorescence intensity readings (S6 Fig).

To examine if TSPANs are involved in another major cellular activity pivotal to pathogenicity, cysteine protease (CP) activity was measured. CP assay was performed using trophozoites of *tspan4*gs, *tspan12*gs, *tspan13*gs, and psAP2 control strains. Both culture medium and cell pellet, corresponding to extracellular (secreted) and intracellular CPs respectively, were collected, after which the CP activity was measured. The activities are shown as percentage relative to the vector control transformant (psAP2 control) (Fig 5D and 5E). We found approximately 50% reduction in the extracellular (secreted) CP activity in *tspan12*gs (50.6 ± 14.6%, *p* = 0.0042) and *tspan13*gs (50.4 ± 2.5%, *p* = 0.0000043) strains, compared to vector control, while no change was observed for *tspan4*gs strain (107.0 ± 21.3%, *p* = 0.6001) (Fig 5D). On the other hand, the intracellular CP activity did not vary significantly in all silenced strains, when compared against mock control. Measured intracellular CP activity of *tspan4*gs, *tspan12*gs, and *tspan13*gs strains were 90.0 ± 7.7%, 78.5 ± 17.0%, and 81.2 ± 29.9%, respectively, to that of the vector control (Fig 5E). Taken together, *tspan12* and *tspan13* gene silencing caused reduction in extracellular, but not intracellular, CP activity, consistent with the premise that TSPAN12 and TSPAN13 are involved in the regulation of CP secretion.

Additionally, based on the potential roles TSPANs played in other organisms, we are interested in exploring whether the previously described regulation of CP activity is attributable to the roles TSPANs play in EVs. We collected crude EV fractions by ultracentrifugation after separating any floating intact cells from the culture medium. The crude EV pellet after 100,000 g ultracentrifugation was collected and permeabilized with 1% Triton-X 100 in PBS. The supernatant of the permeabilized pellet was used to measure the CP activity in crude EV fractions. CP activity in crude EV samples from *tspan4*gs, *tspan12*gs, and *tspan13*gs strains were 423.3 ± 78.0%, 102.5 ± 34.9%, and 90.0 ± 39.4%, respectively (Fig 5F). Interestingly, we found the CP activity in EVs from *tspan4*gs trophozoites drastically increased more than four folds compared with the mock control.

## Discussion

TSPANs play crucial roles in diverse biological processes by engaging with various partners within microdomains [33]. In this study, we demonstrated the constituents and binding profiles of three TSPANs in the TEMs in *E*. *histolytica*. We further demonstrated that some amebic TSPANs participate in virulence mechanisms of this parasite such as adhesion and CP secretion.

Based on multiple proteomic analyses, it is evident that TSPAN4 consistently binds with two other TSPANs, TSPAN12 and TSPAN13, as well as other proteins such as TBP55 and *Eh*interaptin. The interplay among different TSPANs is a common phenomenon conserved in eukaryotes [34]. Although there are 17 TSPANs in *E*. *histolytica*, the specific and exclusive interactions between TSPAN4, TSPAN12, and TSPAN13 stand out amidst the array of other TSPANs. Upon examination of the interacting partners of TSPAN12 and TSPAN13, distinct patterns emerged in comparison to that of TSPAN4. TSPAN12 efficiently bound TSPAN4, whereas TSPAN13 exhibited an inability to pull down both TSPAN4 and TSPAN12. This discrepancy suggests that TSPAN13 may not directly or strongly bind with TSPAN4 and TSPAN12. Additionally, the inability of TSPAN13-HA to pull down other TSPANs raises the possibility that the attached epitope tag could be a contributing factor affecting its binding to other TSPANs.

The number and approximate molecular weight of the *E*. *histolytica* TEM protein complexes seem to vary based on our BN-PAGE analysis of organelle enriched fractions from TSPAN4-HA, TSPAN12-HA, TSPAN13-HA, and TBP55-HA expressing strains (Fig 4). Multiple TEMs were identified in TSPAN12-HA and TBP55-HA expressing stains by anti-HA antibody. The different sizes may be due to different compositions and stoichiometric ratios between TSPANs and other interacting partners. Meanwhile, the absence of a complex in TSPAN13-HA from our BN-PAGE analysis is consistent with the result of MS analysis that TSPAN13-HA was unable to reversely pull-down TSPAN4 and TSPAN12 (S3 Table). This result may indicate that overexpression of TSPAN13, or the epitope tag positioned at the carboxyl terminus, may have a suppressive effect on its complex formation with TSPAN4, TSPAN12 and TBP55, while its binding with *Eh*interaptin seemed unaffected.

In humans, TSPANs play crucial biological roles via their interactions with different single transmembrane-domain binding partners such as integrins, which are involved in cell adhesion and communication between the cell and its external environment. Integrins, composed α and β subunits, bind to extracellular matrix (ECM) proteins or cell surface molecules [31,35–37] Here, we found that *E*. *histolytica* TSPANs also form diverse complexes and bind with various partners, including TBP55 (EHI_001100), also a single transmembrane-domain containing protein previously reported as a mitosomal membrane protein [38]. TBP55 was detected in the TSPAN4-HA, TSPAN12-HA, and TSPAN13-HA co-IP proteomes in a comparatively high QV, which suggests it is a key binding partner of TSPAN4, TSPAN12, and TSPAN13 (Table 3). Aside from a transmembrane domain at the carboxyl terminus, TBP55 also possesses a signal peptide at the amino terminus (S7 Fig) [38]. In the Uniprot database, it is annotated as a von Willebrand factor type A (VWFA)-domain containing protein. VWFA proteins are known to form multiprotein complexes [39], which is also exhibited by TBP55 as indicated by BN-PAGE (Fig 4B). Interestingly, TBP55 was also detected to be expressed significantly higher in non-virulent strain than virulent strain of *E*. *histolytica* [40], suggesting that it may also play a role in the suppression of the parasite’s virulence. The identification of short chain dehydrogenase, steroid 5-alpha reductase, and β-keto acyl reductase implies that TBP55 might play a role in the regulation of fatty acid elongation. Furthermore, the presence of vesicular membrane-associated proteins such as coatomer subunit gamma (EHI_040700) and coated vesicle membrane protein (EHI_096210) suggests that TBP55 could also be involved in vesicular transport between the endoplasmic reticulum (ER) and the cis-Golgi network.

**Table 3:** Summarization of the interactions between major binding partners of TSPAN4 based on the MS analysis results of HA-tagged TSPANs, TBP55 and *Eh*interaptin in co-immunoprecipitation. QV represents quantitative value, normalized with total spectral counts.

**QV(TSPAN4)**		**QV(TSPAN12)**		**QV(TSPAN13)**		**QV(TBP55)**		**QV(*Eh*interaptin)**	
**TSPAN4-HA**	**Mock-HA**	**TSPAN4-HA**	**Mock-HA**	**TSPAN4-HA**	**Mock-HA**	**TSPAN4-HA**	**Mock-HA**	**TSPAN4-HA**	**Mock-HA**
8.7	0	13.1	0	24.0	0	50.1	0	10.9	0
6.1	0	13.8	0	4.6	0	55.3	1.1	1.5	0
28.5	0	13.8	0	28.5	0	210.7	0	7.9	0
**TSPAN12-HA**	**Mock-HA**	**TSPAN12-HA**	**Mock-HA**	**TSPAN12-HA**	**Mock-HA**	**TSPAN12-HA**	**Mock-HA**	**TSPAN12-HA**	**Mock-HA**
3.1	0	4.9	0	2.5	0	8.0	0.4	4.3	1.1
9.8	0	28.2	0	17.4	0	115.0	0	21.7	0
3.1	0	14.1	0	14.1	0	73.6	0	0	0
**TSPAN13-HA**	**Mock-HA**	**TSPAN13-HA**	**Mock-HA**	**TSPAN13-HA**	**Mock-HA**	**TSPAN13-HA**	**Mock-HA**	**TSPAN13-HA**	**Mock-HA**
0	0	0.4	0	17.1	0	0.4	0	22.7	0
0	0	0	0	18.7	0	1.8	0	27.0	0
0	0	0	0	9.2	0	4.9	0	43.6	9.5
**TBP55-HA**	**Mock-HA**	**TBP55-HA**	**Mock-HA**	**TBP55-HA**	**Mock-HA**	**TBP55-HA**	**Mock-HA**	**TBP55-HA**	**Mock-HA**
0	0	21.7	0	96.3	0	85.1	0	0	0
16.2	0	11.3	0	67.5	0	529.1	2.6	0	0
8.9	0	20.0	0	24.5	0	189.2	0	0	0
***Eh*interaptin-HA**	**Mock-HA**	***Eh*interaptin-HA**	**Mock-HA**	***Eh*interaptin-HA**	**Mock-HA**	***Eh*interaptin-HA**	**Mock-HA**	***Eh*interaptin-HA**	**Mock-HA**
0	0	0	0	0	0	0	0	126.0	1.9
0	0	0	0	0	0	0	0	44.9	1.9

The detection of numerous nuclear proteins in our various co-IP experiments using different TSPANs as baits suggests that amebic TSPANs also play a role in nuclear processes. In humans, TSPANs have been implicated in nuclear functions with an example of Tspan8, which was found to carry cholesterol entering the nucleus [41]. Our co-IP MS analysis showed that *Eh*interaptin was consistently identified from transformants expressing TSPAN4-HA, TSPAN12-HA, and TSPAN13-HA (S1–S3 Tables). On the other hand, immunoprecipitation of HA-tagged *Eh*interaptin from the corresponding overexpressing transformant failed to pull down TSPAN4, TSPAN12, and TSPAN13, which may indicate a transient nature of their binding (S4 Table). Interaptin was investigated in a previous research in the free-living amoebae *Dictyostelium discoideum* [42]. *Dd*interaptin (accession number:AF057019) was shown to localize in vesicles of the ER. Both *Dd*interaptin and *Eh*interaptin contain multiple coiled-coil structures based on MARCOIL toolkit prediction (S8A Fig). While *Dd*interaptin possesses an actin-binding domain (ABD) near the amino terminus and a transmembrane helix near the carboxyl terminus, *Eh*interaptin possess neither. Internal repeats in the coiled-coil rod domain are not found in *Eh*interaptin, but when aligned, the putative tyrosine phosphorylation site ‘KKVIDERY’ of *Dd*interaptin has a similar ‘KKRMEELY’ motif in *E*. *histolytica* (S8B Fig). Besides the annotation as interaptin, this protein was predicted to contain a structural maintenance of chromosomes (SMC) domain, which contains long coiled-coil structures and utilizes ATP hydrolysis to drive conformational changes in DNA topology. SMC proteins are essential for chromosome condensation, segregation, and DNA repair and play a vital role in ensuring the stability and proper functioning of the genome [43]. In silico analysis using NCBI’s conserved domain search (https://www.ncbi.nlm.nih.gov/Structure/cdd/wrpsb.cgi) revealed that *Eh*interaptin contains NLS. Furthermore, IFA of *Eh*interaptin-HA showed obvious nuclear, particularly nuclear periphery localization (Fig 3B, top panel). It was previously indicated that the nucleolus is localized at the nuclear periphery in *E*. *histolytica* [44]. Consequently, it is plausible that *Eh*interaptin is situated in the nucleolus of *E*. *histolytica*, given the similarity in the observed signals at the nuclear periphery. In eukaryotes, the SMC complexes are usually built from heterodimers of different SMC subunits that form the basis for cohesin, condensin, and Smc5/6 complexes [45,46]. Since *Eh*interaptin possesses an SMC domain with long coiled-coil structure, it can be one subunit of the SMC complex in *E*. *histolytica*. Also, there is another SMC domain containing protein EHI_152940, which was pulled down by TSPAN4-HA, TSPAN12-HA, and TSPAN13-HA multiple times (S1–S3 Tables). In the MS analysis of *Eh*interaptin-HA co-IP samples, EHI_152940 was also exclusively pulled down in one out of two trials with a very high QV of 56.2. Above these considerations, the data indicate that *Eh*interaptin and EHI_152940 may be potential candidates of SMC subunits in *E*. *histolytica*. A previous study of a SMC family protein indicates that the knock-out of the Smchd1 lead to a ~1.5-fold higher expression of TSPAN 8 (Tspan8) in *Homo sapiens* [47]. This evidence suggests that regulation may exist between TSPANs and SMC-domain containing proteins. The binding between *Eh*interaptin and TSPANs shed light on the transient interactions between SMC complexes and TSPANs in *E*. *histolytica*.

Gal/GalNAc lectin is a surface protein complex that specifically recognizes and binds to Gal/GalNAc on host and bacteria surface molecules including glycoproteins and glycolipids. This lectin plays a pivotal role in the pathogenicity and virulence of *E*. *histolytic*a [48]. A family of Hgl (heavy subunit: EHI_042370, EHI_077500, EHI_133900, EHI_012270, EHI_046650), Igl (intermediate subunit: EHI_006980, EHI_065330), and Lgl (light subunit: EHI_049690, EHI_159870, EHI_058330, EHI_148790, EHI_183400, EHI_135690, EHI_027800) were previously identified [49]. In the TSPANs proteomes, specific members of Hgl, Igl, and Lgl isotypes were identified (S1 File). Such interactions of TSPANs with Gal/GalNac lectins imply that TEMs in *E*. *histolytica* may play a specific role such as adhesion and signal transduction. It is well known that the integrin-TSPAN adhesion complex can modulate integrin signaling in humans [31]. The cytoplasmic domain of Hgl apparently contains a β2 integrin motif, which regulates the adherence and virulence of *E*. *histolytica* [50]. Also, Igl2 (EHI_065330) was identified as a β1 integrin-like fibronectin receptor that assembles a signaling complex similar to those of mammalian cells [51]. In the adhesion assay conducted by utilizing TSPAN12-HA and TSPAN13-HA overexpressing strains, the adhesion capacity was decreased in these overexpressing strains, compared to mock control (Fig 5C). The possible mechanism can be similar with the TSPAN CD82 down-regulating integrin ɑ6-mediated cell adhesion. When CD82 was overexpressed in Du145 prostate cancer cells, the cell surface expression of integrin α6 decreased and the cellular morphogenesis process on Matrigel was abolished [52].

**Fig 6:**
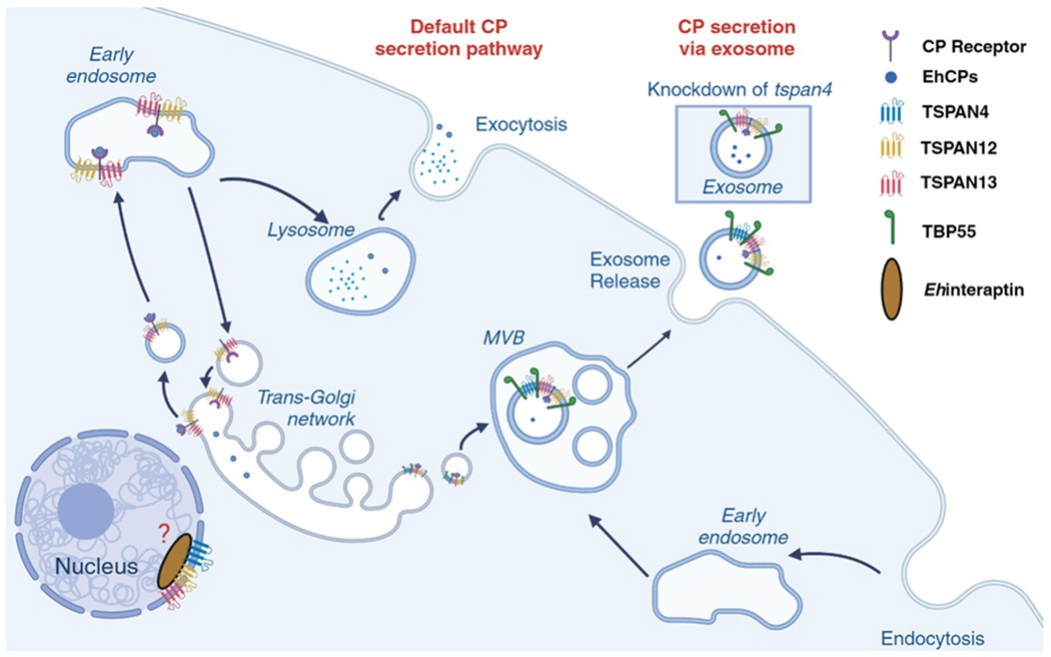
Tetraspanin-mediated regulation of amebic cysteine protease secretion. TSPAN12 and TSPAN13 may participate with cysteine protease (CP) receptor to regulate the secretion of *Eh*CPs via the default CP secretion pathway. TSPAN4 is likely involved in the selection of CPs as exosomal cargo, in a proposed alternative CP secretion via exosomes. TBP55 is a consistent interacting partner of TSPAN4, 12, and 13. Furthermore, the interaction between amebic TSPANs and *Eh*interaptin reflects a potential role of TEMS in the nucleus of this parasite. Created with BioRender.com.

Cysteine proteases have been recognized as key contributors to the pathogenicity of numerous infectious organisms [53]. In recent times, several molecules crucial to the pathogenicity of *E*. *histolytica* have been identified and molecularly characterized [54]. All these molecules, including cysteine proteases, were discovered in both *E*. *histolytica* and *E*. *dispar*, casting doubt on their role in *Entamoeba* pathogenicity. One previous research of two major cysteine proteases uniquely present in *E*. *histolytica* implies that these enzymes are indeed instrumental in the amoeba’s pathogenicity [55]. In a previous research, cysteine protease binding protein 1 (CPBF1, EHI_164800) oversaw the maturation and trafficking of *Eh*CP-A5 (EHI_168240), which is one of the major cysteine proteases in *E*. *histolytica* and responsible for its pathogenicity [56]. Gene silencing of *cpbf1* led to the absence of intracellular mature CP-A5 and enhanced CP-A5 mis-secretion extracellularly [57]. This outcome implies the importance of CPBF1 in the transportation of mature CP-A5 inside the cell, and when CPBF1 was absent, the regular secretion of CP was disrupted. By CP secretion assay, we measure total CP activity in both trophozoite (intracellular) and cell culture supernatant (extracellular). Our data showed that trophozoites of *tspan12*gs and *tspan13*gs secrete less CPs into the extracellular environment (Fig 5D). The decrease in CP extracellular secretion by *tspan12gs* and *tspan13*gs suggests that these TSPANs are involved in the regulation of *Eh*CPs secretion, plausibly instrumental to the stabilization of the function of CPBF1 (Fig 6). Additionally, various *Eh*CPBFs and *Eh*CPs were detected in TSPANs’ proteome especially CPBF1 (S5 Table). Besides measuring extracellular CP activity from culture medium supernatant, we further tested the CP activity in crude EVs, and interestingly found that EVs of *tspan4*gs have a much higher CP activity than the control (Fig 5F). As we know, TSPANs are involved in exosome cargo selection in other organisms [10,58,59]. Thus, it is plausible that TSPAN4 is engaged in cargo selection for *Eh*CPs in the ER or endosomes, packaging them into exosomes, transported via multivesicular bodies, and released extracellularly, in a proposed alternative pathway of amebic CP secretion via exosomes (Fig 6). Although the detailed mechanism of the maturation and traffic of CP-A5 when CPBF1 was silenced remained unknown, the evidence strongly suggests that TSPAN4 plays an important role in controlling the release of *Eh*CPs as EV cargo. The suppression of *Eh*CPs loading into EVs may lead to decreased virulence, consistent with the previous study on TSPAN4 that demonstrated TSPAN4 overexpression caused decreased abscess formation in rodents [22]. We propose that amebic TSPANs are involved in the regulation of CP secretion, a key process in the pathogenesis of *E*. *histolytica*. From our results, it is plausible that TSPAN12 and TSPAN13 are involved in the default CP secretory pathway, whereas TSPAN4 likely regulates CP cargo selection through the exosome secretory pathway.

## Materials and methods

### Organisms, cultivation, and reagents

Trophozoites of *E*. *histolytica* clonal strains HM-1: IMSS cl6 and G3 were cultured axenically in 6 ml screw-capped Pyrex glass tubes in Diamond’s BI-S-33 (BIS) medium [60] and at 35.5°C. The anti-HA 16B12 mouse monoclonal antibody was purchased from Biolegend (San Diego, USA). Lipofectamine, PLUS reagent, and geneticin (G418) were purchased from Invitrogen. CellTracker Green, Orange, and Blue were purchased from Thermo Fisher Scientific (Massachusetts, USA). Restriction enzymes and DNA modifying enzymes were purchased from New England Biolabs (Massachusetts, USA) unless otherwise mentioned. Luria-Bertani (LB) medium was purchased from BD Difco (New Jersey, USA). Other common reagents were purchased from Wako Pure Chemical (Tokyo, Japan), unless otherwise stated.

### Establishment of *E*. *histolytica* transformants

To construct respective plasmids to express TSPAN4 (EHI_075690), TSPAN12 (EHI_091490), TSPAN13 (EHI_107790), TBP55 (EHI_001100) and *Eh*interaptin (EHI_148910) fused with HA tag at the carboxy terminus, DNA fragments corresponding to coding regions were amplified by polymerase chain reaction (PCR) from *E*. *histolytica* cDNA using certain designed primers (See S2 File). The PCR-amplified fragments, and vector plasmids were digested with either *Xho*I and *Xma*I (for N-terminal tagging) or *Bgl*II (for C-terminal tagging), respectively. Amplified fragments were ligated to pEhExHA vector, respectively [61,62]. Ligation between PCR-amplified fragments and vectors were conducted to produce pEhExTSPAN4-HA, pEhExTSPAN12-HA, pEhExTSPAN13-HA, pEhExTBP55-HA, and pEhEx*Eh*interaptin-HA. For G3 strain based antisense small RNA-mediated transcriptional silencing of *tspan4*, *tspan12*, *tspan13*, *tbp55* and psAP2-Gunma vector genes, a fragment measuring 420 base pairs from the gene’s protein-coding section, specifically from the protein’s amino-terminal end, was amplified using PCR from cDNA, this process utilized sense and antisense oligonucleotides that included *Stu*I and *SacI* restriction sites [32,63–65]. Plasmids generated from pEhExHA vector were introduced into the trophozoites of *E*. *histolytica* HM-1: IMSS cl6 strain, whereas plasmids constructed from the psAP2-Gunma vector was introduced into G3 strain by lipofection as described previously [66]. Transformants were initially selected in the presence of 1 μg/ml G418 until the drug concentration was gradually increased to 50 μg/ml for pEhExTSPAN12-HA, pEhExTSPAN13-HA, and 10 μg/ml for remaining strains. Reverse transcriptase PCR was performed to check mRNA levels of *tspan4*, *tspan12*, *tspan13*, *tbp55* gene silenced strains, total RNA was extracted from trophozoites of gene silenced and control strains that were cultivated in the logarithmic phase using TRIZOL reagent (Life Technologies, California, USA). Approximately 1 μg of DNase treated total RNA was used for cDNA synthesis using Superscript III First -Strand Synthesis System (Thermo Fisher Scientific, Massachusetts, USA) with reverse transcriptase and oligo (dT) primer according to the manufacturer’s protocol. ExTaq PCR system was used to amplify DNA from the cDNA template using the primer set written in S2 File.

### Indirect immunofluorescence assay (IFA)

Approximately 5×10^3^ trophozoites in 50 μl BIS were transferred to an 8 mm round well on a slide glass (Matsunami Glass Ind, Osaka, Japan). After 10 minutes incubation in an anaerobic chamber at 35.5°C, removing the medium, cells were fixed with PBS containing 3.7% paraformaldehyde at room temperature for 10 minutes, after fixation, wash the cells with 1x PBS for three times, and subsequently permeabilized with PBS containing 0.2% saponin and 1% bovine serum albumin (BSA) for 10 min each at room temperature. The cells were then reacted with anti-HA mouse monoclonal antibody (1:500), anti-HA rabbit monoclonal antibody (1:200), Hgl 3F4 mouse monoclonal antibody (1: 300) [29,67] or anti-BiP polyclonal antiserum (1,800) [68]. for 1 hour at room temperature. Then the sample was reacted with Alexa Fluor-488 conjugated anti-mouse IgG (1:1,000) antibody, Alexa Fluor-568 conjugated anti-rabbit IgG (1,1,000) antibody (ThermoFisher, Massachusetts, USA) or Hoechst 33342 (diluted into 1 μg/ml) (ThermoFisher, Massachusetts, USA). The images were then captured using LSM 780 confocal microscope and analyzed by ZEN software (Carl-Zeiss, Oberkochen, Germany). The colocalization analysis in ZEN software was utilized. Threshold levels for both green and red channels using single staining controls were set. After which, the same threshold levels were applied to the whole cell images. Using colocalization tool of the Zen software, the calculated coefficient of colocalization was determined.

### Immunoblot analysis

*Entamoeba histolytica* trophozoites expressing TSPAN4-HA grown in the exponential phase were harvested and washed three times with phosphate buffer saline (PBS). After resuspension in lysis buffer [150 mM NaCl, 50 mM Tris-HCl, pH 7.5, 0.1% Triton-X 100, 0.5 mg/ml E-64, and 1x cOmplete Mini protease inhibitor cocktail (Roche, Mannheim, Germany)], the trophozoites were incubated on ice for 30 min, followed by centrifugation at 500 x g for 5 min. Around 30 μg of whole cell lysate were separated on 5–20% SDS-PAGE and followed by electro-transferring onto PVDF membranes. The membranes were incubated in 5% dried skim milk in Tris-Buffered Saline and Tween-20 (TBST; 50 mM Tris-HCl, pH 8.0, 150 mM NaCl, and 0.05% Tween-20) for 30 min at room temperature to block the non-specific binding sites on the membrane. The blots underwent reaction with one of the following primary antibodies, appropriately diluted in TBST as follows: the anti-HA 16B12 monoclonal mouse antibody at a 1:1,000 dilution, the anti-CS1 rabbit polyclonal antiserum [69] at a dilution of 1:1,000, or anti-Vps26-1/2 rabbit polyclonal antiserum at a dilution of 1: 500 [29], all at a temperature of 4°C overnight. Following this, the membranes were washed thrice with TBST and subsequently subjected to further reaction with horseradish peroxidase (HRP)-conjugated anti-mouse or anti-rabbit IgG antiserum, both diluted at a ratio of 1:10,000 and carried out at room temperature for 1 hour. After washing with TBST thrice, the specific proteins were visualized using a chemiluminescence HRP substrate system (Millipore, Massachusetts, USA) and LAS 4000 (Fujifilm Life Science, Cambridge, USA) or ChemiDoc Imaging System (Bio-Rad, USA) as per the manufacturer’s protocol.

### Co-immunoprecipitation (Co-IP)

Approximately 1×10^6^ trophozoites of the amoeba transformants were cultured in a 10-cm dish using BIS medium under a low oxygen environment provided by Anaerocult (Merck) and incubated at 35.5°C for 48 hours. Post medium removal, the amoebae were gently dislodged using cold PBS and chilled on ice for 10 minutes. Following three PBS washes, trophozoites were fixed with DSP solution (Thermo Fisher, Massachusetts, USA). The cells, after a couple of PBS rinses, were lysed in 800 μl of lysis buffer. Post debris clearance by centrifugation at 16,000 *g* at 4°C for 5 minutes, the supernatants were combined with Protein G Sepharose beads (GE Healthcare, Illinois, USA) and incubated at 4°C for an hour. The supernatants, after a brief centrifugation, were transferred to new microtubes containing anti-HA agarose bead-conjugated monoclonal antibodies (Sigma Aldrich) and incubated with gentle mixing at 4°C for 3.5 hours, followed by centrifugation to remove non-binding proteins. The beads underwent three lysis buffer washes and were then incubated in lysis buffer with HA peptide at a 0.2 mg/ml concentration overnight at 4°C to elute the bound proteins.

### Mass spectrometry

Protein sequencing by mass spectrometry was performed by the Mass Spectrometry and Proteomics Core, Johns Hopkins University, Maryland, USA. Each lyophilized protein sample was resuspended in 40μl of 20mM triethylammonium bicarbonate (TEAB), pH 8.0, and reduced with 5μl of 50mM dithiothreitol at 60°C for an hour. After cooling, samples were alkylated with 5μl of 100mM chloroacetamide for 15 minutes in darkness. They were then diluted with 400μl of 9M urea and processed using a 30 kD MWCO spin filter pre-washed with distilled water. Post-washing with urea and TEAB, each sample was treated with 400 ng of enzyme in 300 μl of TEAB and digested overnight at 37°C. The following day, digested peptides were collected post-centrifugation, the filters were rinsed, and the flow-through was combined with the peptides. Peptides were acidified, desalted using an Oasis HLB microelution plate, dried in a speedvac, and reconstituted in 2% acetonitrile with 0.1% formic acid for analysis by a Q-Exactive Plus Hybrid-Quadrupole-Orbitrap mass spectrometer.

### Organellar membrane fractionation

Subcellular fractionation was performed as previously described [70,71], with a few modifications. Six 10-cm dishes of confluent trophozoite cultures in 15 ml BIS medium, for certain transformants, were prepared. After two days of incubation in an anaerobic chamber at 35.5°C, the medium was removed, and 6 ml of cold PBS were added to the dishes. Cells were harvested into 50 ml centrifuge tubes and were centrifuged at 800 *g* for 3 minutes. Pellets were washed 3 times with 1 ml PBS. The wet pellet was weighed and added with the homogenization buffer (SM buffer; 0.25 M sucrose, 10 mM MOPS-KOH, pH 7.2, 0.5 mg/ml E-64, and 1x cOmplete Mini protease inhibitor cocktail) and then homogenized using a glass homogenizer. The cells were homogenized until approximately 20% of the population of trophozoites remained intact. After centrifugation at 5000 *g* for 10 minutes, the supernatant was collected and subjected to fractionation by ultracentrifugation at 100,000 *g* for 60 minutes. The supernatant was collected as the cytosolic fraction. The remaining pellet was resuspended with PBS and utilized in second ultracentrifugation at 100,000 *g* for 60 minutes at 4°C. After centrifugation, the supernatant (cytosolic fraction) was collected, and the pellet (organellar fraction) was resuspended with 100 μl PBS. The mild detergent digitonin was added to samples for solubilization on ice for 30 minutes. The samples were centrifuged at 16,000 *g* for 30 minutes, and then the supernatant was collected as it contained solubilized organellar proteins.

### Blue Native-PAGE

Blue Native-PAGE (BN-Page) was conducted following the protocol from NativePAGE Novex Bis-Tris Gel System kit (Thermo Fisher Scientific, Massachusetts, USA). Subcellular fractionation was performed as described previously [72]. The organelle-enriched fractions permeabilized with 1% digitonin were mixed with NativePage buffer (Thermo Fisher Scientific, Massachusetts, USA) and Coomassie G-250 dye. The BN-Page was performed in XCell SureLock Mini-Cell (Thermo Fisher Scientific, Massachusetts, USA) followed by electro-transferring onto PVDF membranes mentioned above.

### Cysteine protease (CP) secretion assay

CP secretion assay was performed following previous paper with a few modification [57]. Approximately 2.5 × 10^4^ amebic transformants were lysed in 50 μl of transfection medium (Opti-MEM serum reduced medium, pH 6.8, 5.3 mM ascorbic acid, 41.3 mM L-cysteine) by 3 cycles of freezing and thawing, and debris was removed by centrifugation at 14,000 *g* for 5 min. After preincubation of 1 μl of lysate in 74 μl of assay buffer (0.1 M KHPO_4_ pH 6.1, 1 mM EDTA, 2 mM DTT) and 8 μl of supernatant in 68 μl of assay buffer (0.1 M KHPO_4_ pH 6.1, 1 mM EDTA, 2 mM DTT), at room temperature for 10 min, 25 μl of assay buffer containing 2.5 mM benzyloxycarbonyl-L-arginyl-L-arginine-4-methylcoumaryl-7-amide (z-Arg-Arg-MCA, Peptide Institute Inc., Osaka, Japan) was added and fluorescence signal emitted at 505 nm with excitation at 400 nm was recorded for 15 minutes using a SpectraMax Paradigm multimode microplate reader (Molecular Devices, San Jose, CA, USA). z-Arg-Arg-7-amino-4-frifluoromethylcoumarin (AFC) was utilized as a standard. CP activities were expressed in percentage of mock control. The significance of the data was evaluated by unpaired T-test.

### Extracellular vesicles (EVs) isolation

Approximately 50 ml of culture medium of *E*. *histolytica* transformants were first centrifuged at 4°C for 10 minutes at 1,000 g to eliminate intact cells. The supernatant was then filtered through a 0.22 μm membrane using a 50 ml syringe and subsequently concentrated using a 100k MWCO Amicon filter at 4,000 g for 15 minutes. The resultant concentrated cell-free medium was transferred to an ultracentrifuge tube and spun at 100,000 g for 75 minutes at 4°C to precipitate extracellular vesicles, including exosomes. The pellet was washed with 1x PBS, and the centrifugation step was repeated. After discarding the supernatant, the pellet was resuspended in 50 μl of 1x PBS. To lyse the EVs, 1% Triton-X was added, and the mixture was placed on ice for 30 minutes.

### Cell adhesion assay

Transformants were stained with CellTracker Green for 40 minutes at 35.5°C, after washing by PBS, approximately 40,000 cells were seeded into a 96-well collagen-coated plate (Corning biocoat collagen I cellware, USA) and incubated in anaerobic chamber for 40 minutes at 35.5°C. Fluorescence intensity was measured using a SpectraMax Paradigm multimode microplate reader (Molecular Devices, USA) by excitation at 492 nm and emission at 532 nm right after incubation. Fluorescence intensity was re-measured at 492 nm and emission at 532 nm after discarding the medium carefully and one-time washing with warm BI. The percentage of adhesive cells was computed by dividing the final with the initial fluorescence measurements. A standard curve was prepared using stained 7.0 x 10^5^ cells with 2-fold serial dilution.

## Supporting information

S1 Fig Validation of the co-immunoprecipitation of TSPAN12-HA. Representative Immunoblot analysis and silver staining of TSPAN12-HA in co-IP experiments. (A) 5 μl of each fraction collected from co-IP experiments were added into SDS-PAGE and immunoblot analysis using anti-HA antibody. Black arrows indicate TSPAN12-HA expression and red arrows indicate the heavy and light chain of anti-HA antibody. (B) Silver staining was performed by adding 20 μl of eluted fraction of TSPAN12-HA and HA-mock in the co-IP. The black arrows suggest specific bands in the TSPAN12-HA sample.
(TIF)

S2 Fig Validation of the co-immunoprecipitation of TSPAN13-HA. Representative Immunoblot analysis and silver staining of TSPAN13-HA in co-IP experiments. (A) 5 μl of each fraction collected from co-IP experiments were added into SDS-PAGE and immunoblot analysis using anti-HA antibody. Black arrows indicate TSPAN13-HA expression and red arrows indicate the heavy and light chain of anti-HA antibody. (B) Silver staining was performed by adding 30 μl of eluted fraction of TSPAN13-HA and HA-mock in the co-IP. The black arrows suggest specific bands in the TSPAN13-HA sample.
(TIF)

S3 Fig Validation of the co-immunoprecipitation of TBP55-HA. Representative Immunoblot analysis and silver staining of TBP55-HA in co-IP experiments. (A) 5 μl of each fraction collected from co-IP experiments were added into SDS-PAGE and immunoblot analysis using anti-HA antibody. Black arrows indicate TBP55-HA expression and red arrows indicate the heavy and light chain of anti-HA antibody. Green arrows indicate truncated TBP55-HA. (B) Silver staining was performed by adding 20 μl of eluted fraction of TBP55-HA and HA-mock in the co-IP. The black arrows suggest specific bands in the TBP55-HA sample.
(TIF)

S4 Fig Validation of the co-immunoprecipitation of *Eh*interaptin-HA. Representative Immunoblot analysis and silver staining of *Eh*interaptin-HA in co-IP experiments. (A) 5 μl of each fraction collected from co-IP experiments were added into SDS-PAGE and immunoblot analysis using anti-HA antibody. Black arrows indicate *Eh*interaptin-HA expression and red arrows indicate the heavy and light chain of anti-HA antibody. (B) Silver staining was performed by adding 20 μl of eluted fraction of *Eh*interaptin-HA and HA-mock in the Co-IP. The black arrows suggest specific bands in *Eh*interaptin-HA sample.
(TIF)

S5 Fig Representative scatter plots of fluorescence signal distribution to evaluate signal colocalization. Scatter plots of immunofluorescence images from Fig 3A were generated using the “Colocalization” tool of Zen software (Carl Zeiss, Germany) between anti-HA and anti-BiP in TSPAN12-HA (upper panel) and TSPAN13-HA (middle panel) and TBP55-HA (lower panel) respectively. Colocalization coefficient (R; in parentheses) was computed using the same software.
(TIF)

S6 Fig Correlation between *E*. *histolytica* trophozoite cell count and fluorescence intensity. CellTracker Green stained HA-mock transformants were distributed into different wells in collagen-coated 96-well plate in different cell number. Fluorescence intensity was measured after 40 minutes incubation by excitation at 492 nm and emission at 532 nm right after incubation. Triplicate wells were used for each cell number. The linear correlation between cell dye stained trophozoites cell number and fluorescence intensity validate the robustness of the cell adhesion assay.
(TIF)

S7 Fig Domain organization of TSPANs and TBP55 from *Entamoeba histolytica*. TM (Transmembrane domain), SEL (Small extracellular loop), LEL (Large extracellular loop), SP (Signal peptide), red triangles stand for intracellular domains of tetraspanins.
(TIF)

S8 Fig Partial amino acids sequence alignment between *Dd*interaptin and *Eh*interaptin and Coiled-coil (CC) structure prediction for *Eh*interaptin (EHI_148910). (A) Multiple coiled-coil structures were predicted in *Eh*interaptin (EHI_148910) by MARCOIL toolkit (https://toolkit.tuebingen.mpg.de/tools/marcoil) which is a HMM model for coiled-coil domains. (B) Multiple amino acids sequence alignment between Ddinteraptin (AF057019) and Ehinteraptin (EHI_148910) were built by clustal W algorithm. The putative tyrosine phosphorylation site ‘KKVIDERY’ in Ddinteraptin is shown in blue, while the similar motif in Ehinteraptin ‘KKRMEELY’ is shown in red.
(TIF)

S9 Fig Alignment of TSPANs in *Entamoeba histolytica*. Multiple amino acids sequence alignment of *Entamoeba histolytica* TSPAN4(EHI_075690), TSPAN12(EHI_091490), TSPAN13(EHI_107790) was established by clustal W algorithm (https://www.genome.jp/tools-bin/clustalw). The highly conserved CCG motifs and the following conserved tryptophan and lysine residues located at large extracellular loops are shown in red font.
(TIF)

S1 Table Mass-spectrometry results of HA-tagged TSPAN4 co-immunoprecipitation. Experimental details referred to Table 1 description but with the bait protein HA-tagged TSPAN4. The list order is sorted by frequency of identification firstly and mean of quantitative value secondly.
(DOCX)

S2 Table Mass-spectrometry results of HA-tagged TSPAN12 co-immunoprecipitation. Experimental details referred to Table 1 description but with the bait protein HA-tagged TSPAN12. The list order is sorted by frequency of identification firstly and mean of quantitative value secondly.
(DOCX)

S3 Table Mass-spectrometry results of HA-tagged TSPAN13 co-immunoprecipitation. Experimental details referred to Table 1 description but with the bait protein HA-tagged TSPAN13. The list order is sorted by frequency of identification firstly and mean of quantitative value secondly.
(DOCX)

S4 Table Mass-spectrometry results of HA-tagged *Eh*interaptin co-immunoprecipitation. Experimental details referred to Table 1 description but with the bait protein HA-tagged *Eh*interaptin. The Co-IP and MS analysis were conducted twice independently. The list order is sorted by frequency of identification firstly and mean of quantitative value secondly.
(DOCX)

S5 Table *Eh*CPBFs and *Eh*CPs proteins pulled-down by amebic TSPANs and their binding proteins. *Eh*CPBFs and *Eh*CPs proteins found in TSPANs-HA and TBP55-HA proteome. The upper panel of TSPAN4-HA, TSPAN12-HA, TSPAN13-HA, TBP55-HA indicate the number of independent trials the protein was pulled down by the certain bait protein. The lower panel suggests the mean QV that the protein detected in TSPANs or TBP55, the number inside bracket suggests mean QV that the protein detected in the mock control. ND, no detection.
(DOCX)

S6 Table Percentage of amino acid identity among TSPAN4, TSPAN12 and TSPAN13. The alignment was conducted by ClustalW multiple sequence alignment toolkit.
(DOCX)

S1 File Pull down list for all mass-spectrometry experiments. (XLSX)

S2 File Primer sequences used in experiments. (XLSX)

S1 Dataset Data from all independent trials of adhesion assay (in Fig 5B–5C) and CP activity assay (in Fig 5D–5F). (XLSX)

## References

[1] Copeland BT, Bowman MJ, Ashman LK. Genetic ablation of the tetraspanin CD151 reduces spontaneous metastatic spread of prostate cancer in the TRAMP model. Mol Cancer Res. 2013;11(1):95–105. doi: 10.1158/1541-7786.MCR-12-0468 23131993

[2] Copeland BT, Bowman MJ, Boucheix C, Ashman LK. Knockout of the tetraspanin Cd9 in the TRAMP model of de novo prostate cancer increases spontaneous metastases in an organ-specific manner. Int J Cancer. 2013;133(8):1803–12. doi: 10.1002/ijc.28204 23575960

[3] Charrin S, Jouannet S, Boucheix C, Rubinstein E. Tetraspanins at a glance. J Cell Sci. 2014;127(Pt 17):3641–8. doi: 10.1242/jcs.154906 25128561

[4] Deng X, Li Q, Hoff J, Novak M, Yang H, Jin H, et al. Integrin-associated CD151 drives ErbB2-evoked mammary tumor onset and metastasis. Neoplasia. 2012;14(8):678–89. doi: 10.1593/neo.12922 22952421 PMC3431176

[5] Jones EL, Demaria MC, Wright MD. Tetraspanins in cellular immunity. Biochem Soc Trans. 2011;39(2):506–11. doi: 10.1042/BST0390506 21428929

[6] van Spriel AB, Figdor CG. The role of tetraspanins in the pathogenesis of infectious diseases. Microbes Infect. 2010;12(2):106–12. doi: 10.1016/j.micinf.2009.11.001 19896556

[7] Piratae S, Tesana S, Jones MK, Brindley PJ, Loukas A, Lovas E, et al. Molecular characterization of a tetraspanin from the human liver fluke, Opisthorchis viverrini. PLoS Negl Trop Dis. 2012;6(12):e1939. doi: 10.1371/journal.pntd.0001939 23236532 PMC3516575

[8] Gobert GN, Tran MH, Moertel L, Mulvenna J, Jones MK, McManus DP, et al. Transcriptional changes in *Schistosoma mansoni* during early schistosomula development and in the presence of erythrocytes. PLoS Negl Trop Dis. 2010;4(2):e600. doi: 10.1371/journal.pntd.0000600 20161728 PMC2817720

[9] Mekonnen GG, Pearson M, Loukas A, Sotillo J. Extracellular vesicles from parasitic helminths and their potential utility as vaccines. Expert Rev Vaccines. 2018;17(3):197–205. doi: 10.1080/14760584.2018.1431125 29353519

[10] Twu O, de Miguel N, Lustig G, Stevens GC, Vashisht AA, Wohlschlegel JA, et al. *Trichomonas vaginalis* exosomes deliver cargo to host cells and mediate host∶parasite interactions. PLoS Pathog. 2013;9(7):e1003482. doi: 10.1371/journal.ppat.1003482 23853596 PMC3708881

[11] de Miguel N, Riestra A, Johnson PJ. Reversible association of tetraspanin with Trichomonas vaginalis flagella upon adherence to host cells. Cell Microbiol. 2012;14(12):1797–807. doi: 10.1111/cmi.12003 22882837 PMC4437513

[12] Yáñez-Mó M, Barreiro O, Gordon-Alonso M, Sala-Valdés M, Sánchez-Madrid F. Tetraspanin-enriched microdomains: a functional unit in cell plasma membranes. Trends Cell Biol. 2009;19(9):434–46. doi: 10.1016/j.tcb.2009.06.004 19709882

[13] Klein-Soyer C, Azorsa DO, Cazenave JP, Lanza F. CD9 Participates in endothelial cell migration during in vitro wound repair. Arterioscler Thromb Vasc Biol. 2000;20(2):360–9. doi: 10.1161/01.atv.20.2.360 10669631

[14] Peñas PF, García-Díez A, Sánchez-Madrid F, Yáñez-Mó M. Tetraspanins are localized at motility-related structures and involved in normal human keratinocyte wound healing migration J Invest Dermatol. 2000;114(6):1126–35. doi: 10.1046/j.1523-1747.2000.00998.x 10844555

[15] Boucheix C, Rubinstein E. Tetraspanins. Cell Mol Life Sci. 2001;58(9):1189–205. doi: 10.1007/PL00000933 11577978 PMC11337403

[16] Berditchevski F. Complexes of tetraspanins with integrins: more than meets the eye. J Cell Sci. 2001;114(Pt 23):4143–51. doi: 10.1242/jcs.114.23.4143 11739647

[17] Hemler ME. Tetraspanin functions and associated microdomains. Nat Rev Mol Cell Biol. 2005;6(10):801–11. doi: 10.1038/nrm1736 16314869

[18] Lozano R, Naghavi M, Foreman K, Lim S, Shibuya K, Aboyans V, et al. Global and regional mortality from 235 causes of death for 20 age groups in 1990 and 2010: a systematic analysis for the Global Burden of Disease Study 2010. Lancet. 2012;380(9859):2095–128. doi: 10.1016/S0140-6736(12)61728-0 23245604 PMC10790329

[19] Haque R, Huston CD, Hughes M, Houpt E, Petri WA. Amebiasis. N Engl J Med. 2003;348(16):1565–73. doi: 10.1056/NEJMra022710 12700377

[20] Stanley SL. Amoebiasis. Lancet. 2003;361(9362):1025–34. doi: 10.1016/S0140-6736(03)12830-9 12660071

[21] Tomii K, Santos HJ, Nozaki N. Genome-wide analysis of known and potential tetraspanins in *Entamoeba histolytica*. Genes (Basel). 2019;10(11):885. doi: 10.3390/genes10110885 31684194 PMC6895871

[22] Meyer M, Fehling H, Matthiesen J, Lorenzen S, Schuldt K, Bernin H, et al. Overexpression of differentially expressed genes identified in non-pathogenic and pathogenic *Entamoeba histolytica* clones allow identification of new pathogenicity factors involved in amoebic liver abscess formation. PLoS Pathog. 2016;12(8):e1005853. doi: 10.1371/journal.ppat.1005853 27575775 PMC5004846

[23] Castellanos-Castro S, Bolaños J, Orozco E. Lipids in *Entamoeba histolytica*: host-dependence and virulence factors. Front Cell Infect Microbiol. 2020;10:75. doi: 10.3389/fcimb.2020.00075 32211340 PMC7075943

[24] Marquay Markiewicz J, Syan S, Hon CC, Weber C, Faust D, Guillen N. A proteomic and cellular analysis of uropods in the pathogen *Entamoeba histolytica*. PLoS Negl Trop Dis. 2011;5(4):e1002. doi: 10.1371/journal.pntd.0001002 21483708 PMC3071361

[25] Little KD, Hemler ME, Stipp CS. Dynamic regulation of a GPCR-tetraspanin-G protein complex on intact cells: central role of CD81 in facilitating GPR56-Gαq/11 association. Mol Biol Cell. 2004;15(5):2375–87. doi: 10.1091/mbc.e03-12-0886 15004227 PMC404030

[26] Strambio-De-Castillia C, Niepel M, Rout MP. The nuclear pore complex: bridging nuclear transport and gene regulation. Nat Rev Mol Cell Biol. 2010;11(7):490–501. doi: 10.1038/nrm2928 20571586

[27] Gomez-Cavazos JS, Hetzer MW. The nucleoporin gp210/Nup210 controls muscle differentiation by regulating nuclear envelope/ER homeostasis. J Cell Biol. 2015;208(6):671–81. doi: 10.1083/jcb.201410047 25778917 PMC4362455

[28] Solmaz SR, Blobel G, Melčák I. Ring cycle for dilating and constricting the nuclear pore. Proc Natl Acad Sci USA. 2013;110(15):5858–63. doi: 10.1073/pnas.1302655110 23479651 PMC3625290

[29] Nakada-Tsukui K, Saito-Nakano Y, Ali V, Nozaki T. A retromerlike complex is a novel Rab7 effector that is involved in the transport of the virulence factor cysteine protease in the enteric protozoan parasite *Entamoeba histolytica*. Mol Biol Cell. 2005;16(11):5294–303. doi: 10.1091/mbc.e05-04-0283 16120649 PMC1266427

[30] Martínez-Valencia D, Bañuelos C, García-Rivera G, Talamás-Lara D, Orozco E. The *Entamoeba histolytica* Vps26 (EhVps26) retromeric protein is involved in phagocytosis: Bioinformatic and experimental approaches. PLoS One. 2024;19(8):e0304842. doi: 10.1371/journal.pone.0304842 39116045 PMC11309391

[31] Berditchevski F, Odintsova E. Characterization of integrin—tetraspanin adhesion complexes. J Cell Biol. 1999;146(2):477–92. doi: 10.1083/jcb.146.2.477 10427099 PMC2156181

[32] Mirelman D, Anbar M, Bracha R. Epigenetic transcriptional gene silencing in *Entamoeba histolytica*. 2008;60(9):598–604. doi: 10.1002/iub.96 18493998

[33] van Deventer SJ, Dunlock VME, van Spriel AB. Molecular interactions shaping the tetraspanin web. Biochem Soc Trans. 2017;45(3):741–750. doi: 10.1042/BST20160284 28620035

[34] Levy S, Shoham T. The tetraspanin web modulates immune-signalling complexes. Nat Rev Immunol. 2005;5(2):136–48. doi: 10.1038/nri1548 15688041

[35] Takawale A, Zhang P, Patel VB, Wang X, Oudit G, Kassiri Z. Tissue inhibitor of matrix metalloproteinase-1 promotes myocardial fibrosis by mediating CD63–integrin β1 interaction. Hypertension. 2017;69(6):1092–1103. doi: 10.1161/HYPERTENSIONAHA.117.09045 28373589

[36] Tugues S, Honjo S, König C, Padhan N, Kroon J, Gualandi L, et al. Tetraspanin CD63 promotes vascular endothelial growth factor receptor 2-β1 integrin complex formation, thereby regulating activation and downstream signaling in endothelial cells in vitro and in vivo. J Biol Chem. 2013;288(26):19060–71. doi: 10.1074/jbc.M113.468199 23632027 PMC3696679

[37] Peddibhotla SSD, Brinkmann BF, Kummer D, Tuncay H, Nakayama M, Adams RH, et al. Tetraspanin CD9 links junctional adhesion molecule-A to αvβ3 integrin to mediate basic fibroblast growth factor—specific angiogenic signaling. Mol Biol Cell. 2013;24(7):933–44. doi: 10.1091/mbc.E12-06-0481 23389628 PMC3608503

[38] Santos HJ, Imai K, Hanadate Y, Fukasawa Y, Oda T, Mi-ichi F, et al. Screening and discovery of lineage-specific mitosomal membrane proteins in *Entamoeba histolytica*. Mol Biochem Parasitol. 2016;209(1–2):10–17. doi: 10.1016/j.molbiopara.2016.01.001 26792249

[39] Whittaker CA, Hynes RO. Distribution and evolution of von Willebrand/integrin A domains: widely dispersed domains with roles in cell adhesion and elsewhere. Mol Biol Cell. 2002;13(10):3369–87. doi: 10.1091/mbc.e02-05-0259 12388743 PMC129952

[40] Ng YL, Olivos-García A, Lim TK, Noordin R, Lin Q, Othman N. *Entamoeba histolytica*: quantitative proteomics analysis reveals putative virulence-associated differentially abundant membrane proteins. Am J Trop Med Hyg. 2018;99(6):1518–1529. doi: 10.4269/ajtmh.18-0415 30298805 PMC6283474

[41] Huang Y, Li J, Du W, Li S, Li Y, Qu H, et al. Nuclear translocation of the 4-pass transmembrane protein Tspan8. Cell Res. 2021;31(11):1218–1221. doi: 10.1038/s41422-021-00522-9 34099887 PMC8563794

[42] Rivero F, Kuspa A, Brokamp R, Matzner M, Noegel AA. Interaptin, an actin-binding protein of the α-actinin superfamily in *Dictyostelium discoideum*, is developmentally and cAMP-regulated and associates with intracellular membrane compartments. J Cell Biol. 1998;142(3):735–50. doi: 10.1083/jcb.142.3.735 9700162 PMC2148174

[43] Hassler M, Shaltiel IA, Haering CH. Towards a unified model of SMC complex function. Curr Biol. 2018;28(21):R1266–R1281. doi: 10.1016/j.cub.2018.08.034 30399354 PMC6850909

[44] Jhingan GD, Panigrahi SK, Bhattacharya A, Bhattacharya S. The nucleolus in *Entamoeba histolytica* and *Entamoeba invadens* is located at the nuclear periphery. Mol Biochem Parasitol. 2009;167(1):72–80. doi: 10.1016/j.molbiopara.2009.04.011 19416742

[45] Uhlmann F. SMC complexes: from DNA to chromosomes. Nat Rev Mol Cell Biol. 2016;17(7):399–412. doi: 10.1038/nrm.2016.30 27075410

[46] van Ruiten MS, Rowland BD. SMC complexes: universal DNA looping machines with distinct regulators. Trends Genet. 2018;34(6):477–487. doi: 10.1016/j.tig.2018.03.003 29606284

[47] Midic U, Vincent KA, Wang K, Lokken A, Severance AL, Ralston A, et al. Novel key roles for structural maintenance of chromosome flexible domain containing 1 (Smchd1) during preimplantation mouse development. Mol Reprod Dev. 2018;85(7):635–648. doi: 10.1002/mrd.23001 29900695 PMC6361378

[48] Mann BJ. Structure and function of the *Entamoeba histolytica* Gal/GalNAc lectin. Int Rev Cytol. 2002:216:59–80. doi: 10.1016/s0074-7696(02)16003-7 12049210

[49] Weedall GD, Sherrington J, Paterson S, Hall N. Evidence of gene conversion in genes encoding the Gal/GalNac lectin complex of *Entamoeba*. PLoS Negl Trop Dis. 2011;5(6):e1209. doi: 10.1371/journal.pntd.0001209 21738808 PMC3125142

[50] Vines RR, Ramakrishnan G, Rogers JB, Lockhart LA, Mann BJ, Petri WA. Regulation of adherence and virulence by the *Entamoeba histolytica* lectin cytoplasmic domain, which contains a β2 integrin motif. Mol Biol Cell. 1998;9(8):2069–79. doi: 10.1091/mbc.9.8.2069 9693367 PMC25460

[51] Flores-Robles D, Rosales C, Rosales-Encina JL, Talamás-Rohana P. *Entamoeba histolytica*: a β1 integrin-like fibronectin receptor assembles a signaling complex similar to those of mammalian cells. Exp Parasitol. 2003;103(1–2):8–15. doi: 10.1016/s0014-4894(03)00062-6 12810041

[52] He B, Liu L, Cook GA, Grgurevich S, Jennings LK, Zhang XA. Tetraspanin CD82 attenuates cellular morphogenesis through down-regulating integrin α6-mediated cell adhesion. Journal of Biological Chemistry. 2005;280(5):3346–54. doi: 10.1074/jbc.M406680200 15557282

[53] Sajid M, McKerrow JH. Cysteine proteases of parasitic organisms. Mol Biochem Parasitol. 2002;120(1):1–21. doi: 10.1016/s0166-6851(01)00438-8 11849701

[54] Rawat A, Roy M, Jyoti A, Kaushik S, Verma K, Srivastava VK. Cysteine proteases: Battling pathogenic parasitic protozoans with omnipresent enzymes. Microbiol Res. 2021;249:126784. doi: 10.1016/j.micres.2021.126784 33989978

[55] Bruchhaus I, Jacobs T, Leippe M, Tannich E. *Entamoeba histolytica* and *Entamoeba dispar*: differences in numbers and expression of cysteine proteinase genes. Mol Microbiol. 1996;22(2):255–63. doi: 10.1046/j.1365-2958.1996.00111.x 8930910

[56] Irmer H, Tillack M, Biller L, Handal G, Leippe M, Roeder T, et al. Major cysteine peptidases of *Entamoeba histolytica* are required for aggregation and digestion of erythrocytes but are dispensable for phagocytosis and cytopathogenicity. Mol Microbiol. 2009;72(3):658–67. doi: 10.1111/j.1365-2958.2009.06672.x 19426210

[57] Nakada-Tsukui K, Tsuboi K, Furukawa A, Yamada Y, Nozaki T. A novel class of cysteine protease receptors that mediate lysosomal transport. Cell Microbiol. 2012;14(8):1299–317. doi: 10.1111/j.1462-5822.2012.01800.x 22486861 PMC3465781

[58] MacDonald C, Payne JA, Aboian M, Smith W, Katzmann DJ, Piper RC. A family of tetraspanins organizes cargo for sorting into multivesicular bodies. Dev Cell. 2015;33(3):328–42. doi: 10.1016/j.devcel.2015.03.007 25942624 PMC4421094

[59] Rana S, Zöller M. Exosome target cell selection and the importance of exosomal tetraspanins: a hypothesis. Biochem Soc Trans. 2011;39(2):559–62. doi: 10.1042/BST0390559 21428939

[60] Diamond LS, Harlow DR, Cunick CC. A new medium for the axenic cultivation of Entamoeba histolytica and other *Entamoeba*. Trans R Soc Trop Med Hyg. 1978;72(4):431–2. doi: 10.1016/0035-9203(78)90144-x 212851

[61] Nakada-Tsukui K, Okada H, Mitra BN, Nozaki T. Phosphatidylinositol-phosphates mediate cytoskeletal reorganization during phagocytosis via a unique modular protein consisting of RhoGEF/DH and FYVE domains in the parasitic protozoon *Entamoeba histolytica*. Cell Microbiol. 2009;11(10):1471–91. doi: 10.1111/j.1462-5822.2009.01341.x 19496789

[62] Somlata, Nakada-Tsukui K, Nozaki T. AGC family kinase 1 participates in trogocytosis but not in phagocytosis in *Entamoeba histolytica*. Nat Commun. 2017;8(1):101. doi: 10.1038/s41467-017-00199-y 28740237 PMC5524646

[63] Bracha R, Nuchamowitz Y, Mirelman D. Transcriptional silencing of an amoebapore gene in *Entamoeba histolytica*: Molecular analysis and effect on pathogenicity. Eukaryot Cell. 2003;2(2):295–305. doi: 10.1128/EC.2.2.295-305.2003 12684379 PMC154849

[64] Bracha R, Nuchamowitz Y, Mirelman D. Amoebapore is an important virulence factor of *Entamoeba histolytica*. J Biosci. 2002;27(6):579–87. doi: 10.1007/BF02704851

[65] Bracha R, Nuchamowitz Y, Anbar M, Mirelman D. Transcriptional silencing of multiple genes in trophozoites of *Entamoeba histolytica*. PLoS Pathog. 2006;2(5):e48. doi: 10.1371/journal.ppat.0020048 16733544 PMC1464398

[66] Mi-ichi F, Makiuchi T, Furukawa A, Sato D, Nozaki T. Sulfate activation in mitosomes plays an important role in the proliferation of *Entamoeba histolytica*. PLoS Negl Trop Dis. 2011;5(8):e1263. doi: 10.1371/journal.pntd.0001263 21829746 PMC3149026

[67] Petri WA, Mann BJ. Molecular mechanisms of invasion by *Entamoeba histolytica*. Semin Cell Biol. 1993;4(5):305–13. doi: 10.1006/scel.1993.1037 7504959

[68] Hanadate Y, Saito-Nakano Y, Nakada-Tsukui K, Nozaki T. Endoplasmic reticulum-resident Rab8A GTPase is involved in phagocytosis in the protozoan parasite *Entamoeba histolytica*. Cell Microbiol. 2016;18(10):1358–73. doi: 10.1111/cmi.12570 26807810 PMC5071775

[69] Nozaki T, Asai T, Kobayashi S, Ikegami F, Noji M, Saito K, et al. Molecular cloning and characterization of the genes encoding two isoforms of cysteine synthase in the enteric protozoan parasite *Entamoeba histolytica*. Mol Biochem Parasitol. 1998;97(1–2):33–44. doi: 10.1016/s0166-6851(98)00129-7 9879885

[70] Makiuchi T, Mi-ichi F, Nakada-Tsukui K, Nozaki T. Novel TPR-containing subunit of TOM complex functions as cytosolic receptor for *Entamoeba* mitosomal transport. Sci Rep. 2013;3:1129. doi: 10.1038/srep01129 23350036 PMC3553487

[71] Santos HJ, Hanadate Y, Imai K, Watanabe H, Nozaki T. *Entamoeba histolytica* EHD1 Is involved in mitosome-endosome contact. mBio. 2022;13(2):e0384921. doi: 10.1128/mbio.03849-21 35404118 PMC9040739

[72] Makiuchi T, Santos HJ, Tachibana H, Nozaki T. Hetero-oligomer of dynamin-related proteins participates in the fission of highly divergent mitochondria from *Entamoeba histolytica*. Sci Rep. 2017;7(1):13439. doi: 10.1038/s41598-017-13721-5 29044162 PMC5647421

