# Supplementary figures and images for: Tetraspanin-enriched microdomains play an important role in pathogenesis in the protozoan parasite Entamoeba histolytica
Source: PLoS Pathog. 2024 Oct 3;20(10):e1012151. doi: 10.1371/journal.ppat.1012151 (PMC11478834; doi:10.1371/journal.ppat.1012151)

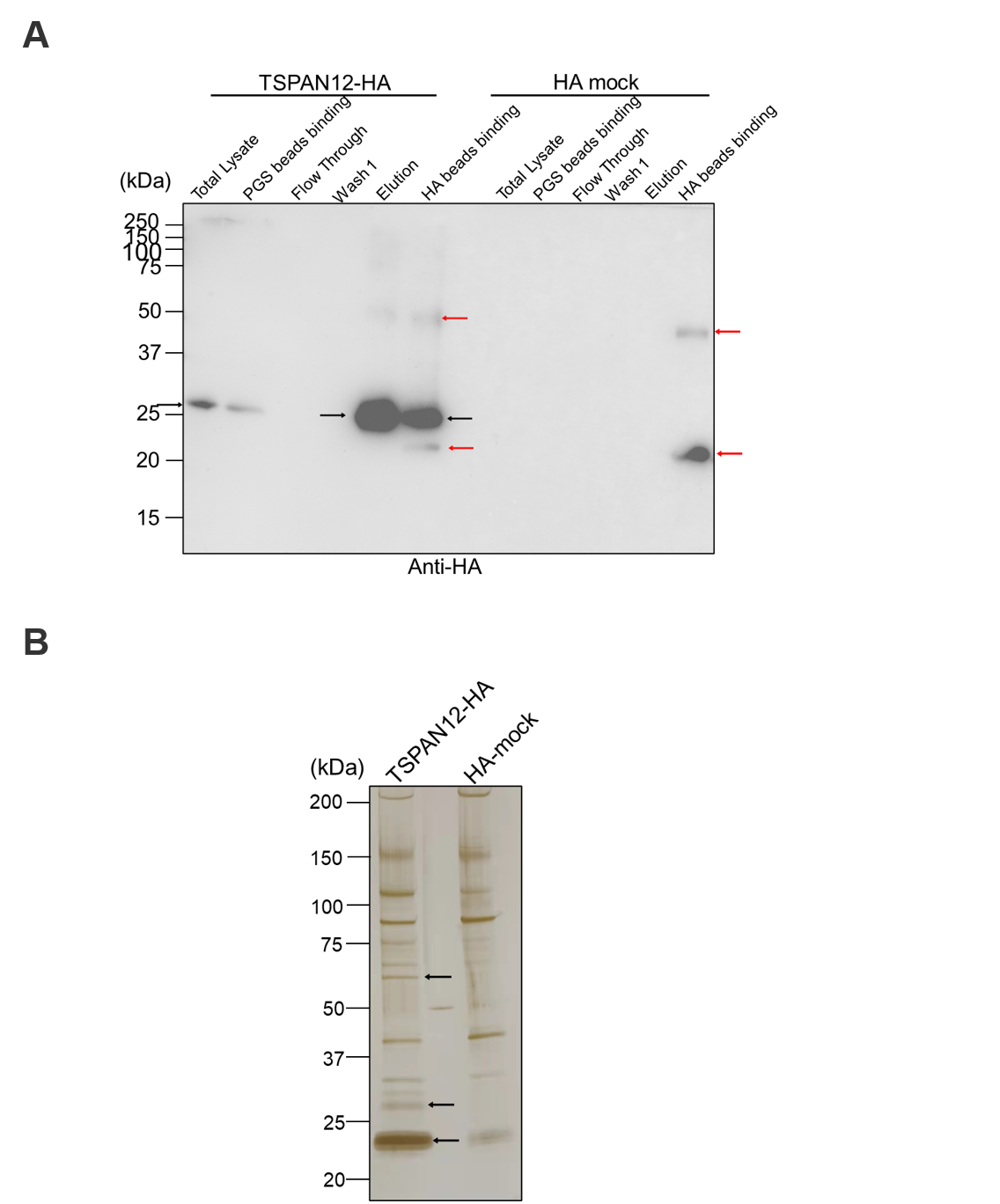

Supplement: S1 Fig — Representative Immunoblot analysis and silver staining of TSPAN12-HA in co-IP experiments. (A) 5 μl of each fraction collected from co-IP experiments were added into SDS-PAGE and immunoblot analysis using anti-HA antibody. Black arrows indicate TSPAN12-HA expression and red arrows indicate the heavy and light chain of anti-HA antibody. (B) Silver staining was performed by adding 20 μl of eluted fraction of TSPAN12-HA and HA-mock in the co-IP. The black arrows suggest specific bands in the TSPAN12-HA sample. (TIF) [file ppat.1012151.s001.tif]

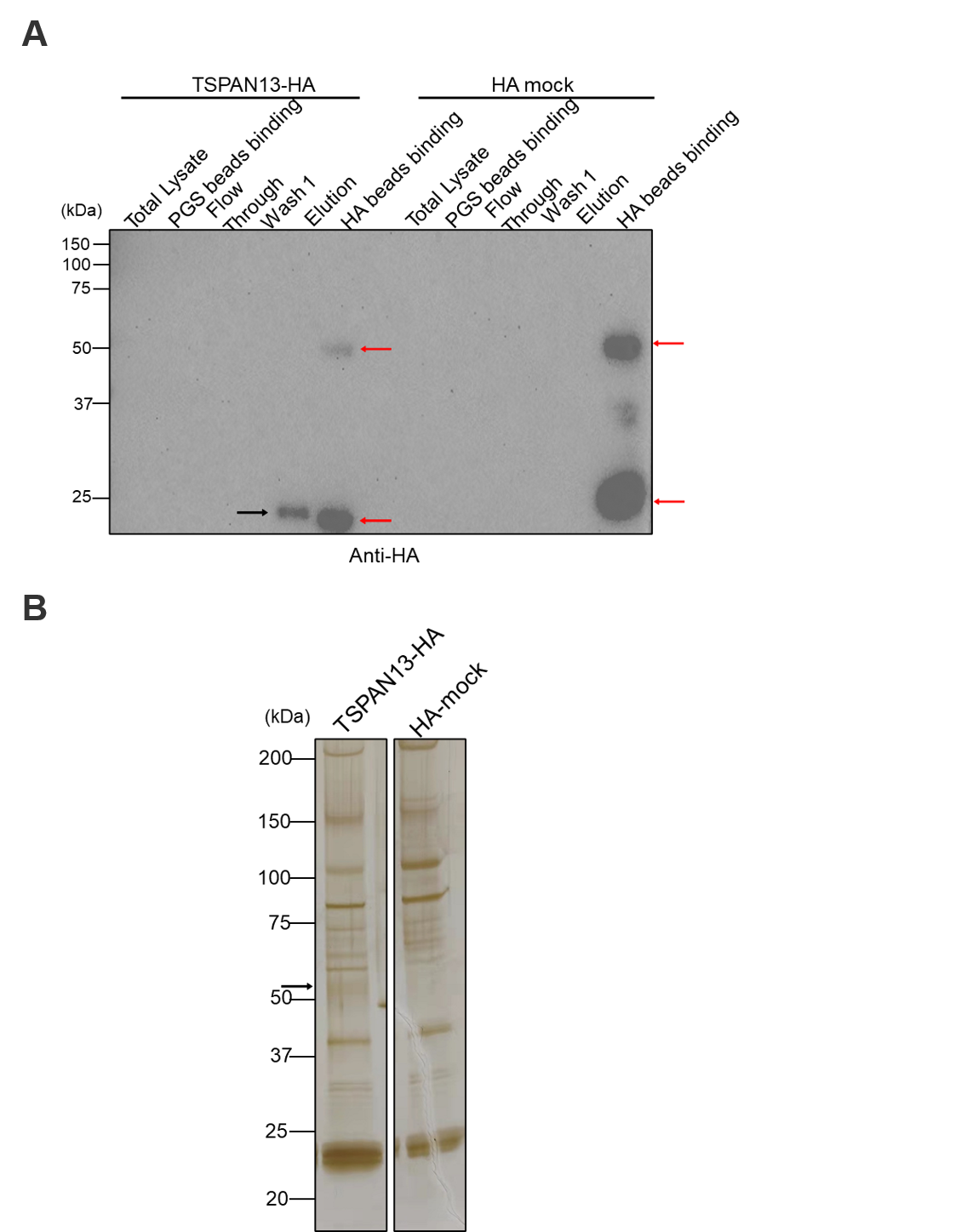

Supplement: S2 Fig — Representative Immunoblot analysis and silver staining of TSPAN13-HA in co-IP experiments. (A) 5 μl of each fraction collected from co-IP experiments were added into SDS-PAGE and immunoblot analysis using anti-HA antibody. Black arrows indicate TSPAN13-HA expression and red arrows indicate the heavy and light chain of anti-HA antibody. (B) Silver staining was performed by adding 30 μl of eluted fraction of TSPAN13-HA and HA-mock in the co-IP. The black arrows suggest specific bands in the TSPAN13-HA sample. (TIF) [file ppat.1012151.s002.tif]

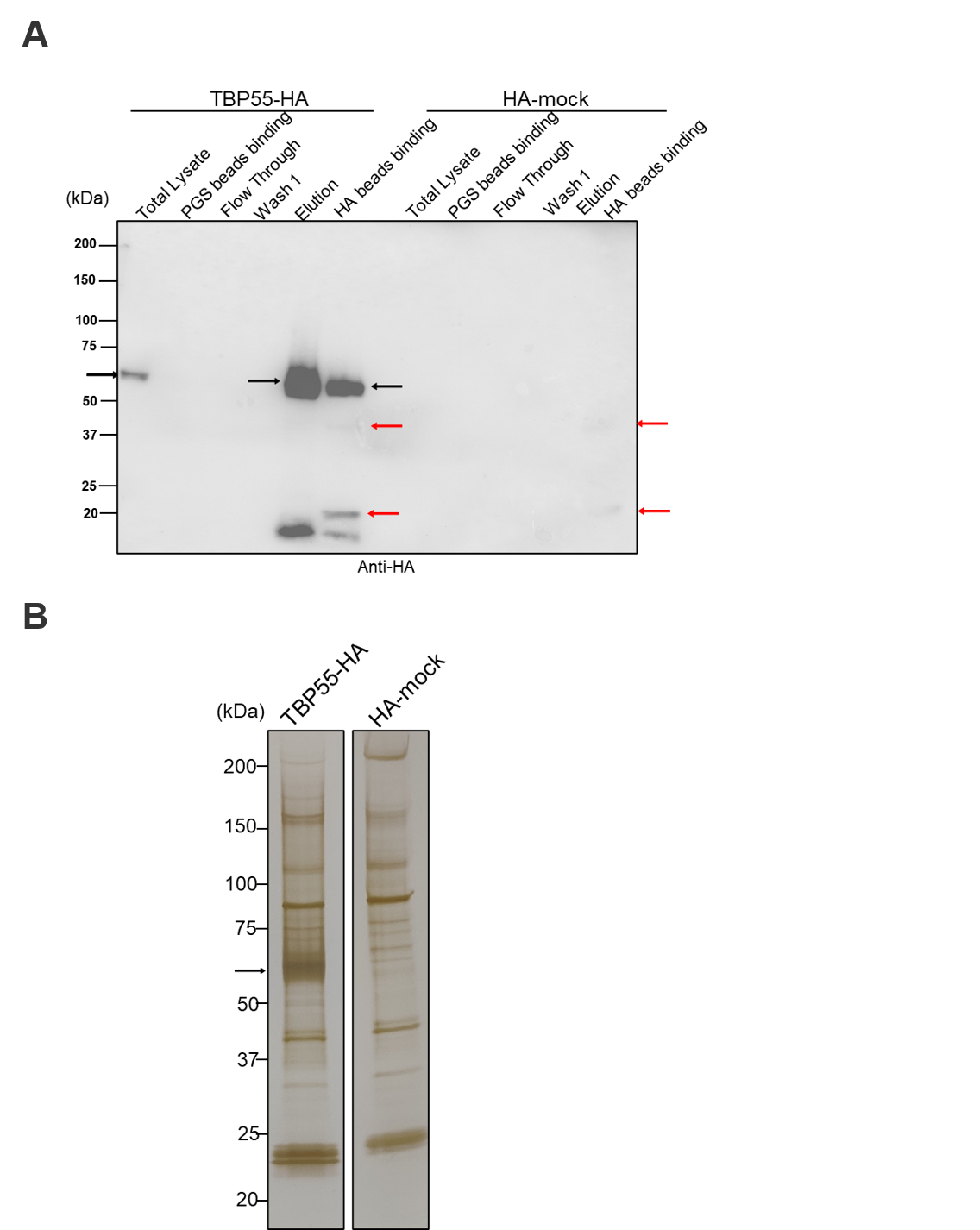

Supplement: S3 Fig — Representative Immunoblot analysis and silver staining of TBP55-HA in co-IP experiments. (A) 5 μl of each fraction collected from co-IP experiments were added into SDS-PAGE and immunoblot analysis using anti-HA antibody. Black arrows indicate TBP55-HA expression and red arrows indicate the heavy and light chain of anti-HA antibody. Green arrows indicate truncated TBP55-HA. (B) Silver staining was performed by adding 20 μl of eluted fraction of TBP55-HA and HA-mock in the co-IP. The black arrows suggest specific bands in the TBP55-HA sample. (TIF) [file ppat.1012151.s003.tif]

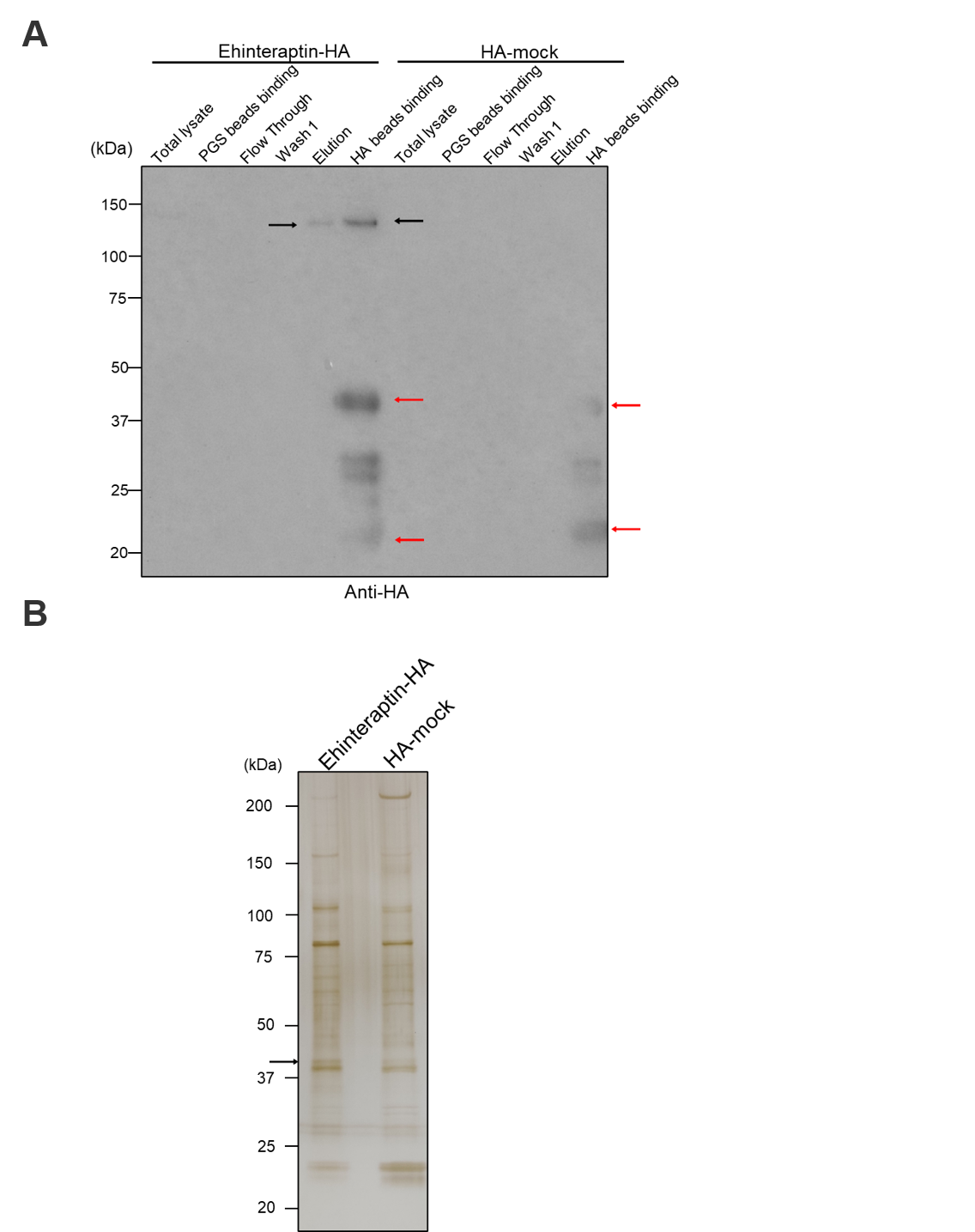

Supplement: S4 Fig — Representative Immunoblot analysis and silver staining of Ehinteraptin-HA in co-IP experiments. (A) 5 μl of each fraction collected from co-IP experiments were added into SDS-PAGE and immunoblot analysis using anti-HA antibody. Black arrows indicate Ehinteraptin-HA expression and red arrows indicate the heavy and light chain of anti-HA antibody. (B) Silver staining was performed by adding 20 μl of eluted fraction of Ehinteraptin-HA and HA-mock in the Co-IP. The black arrows suggest specific bands in Ehinteraptin-HA sample. (TIF) [file ppat.1012151.s004.tif]

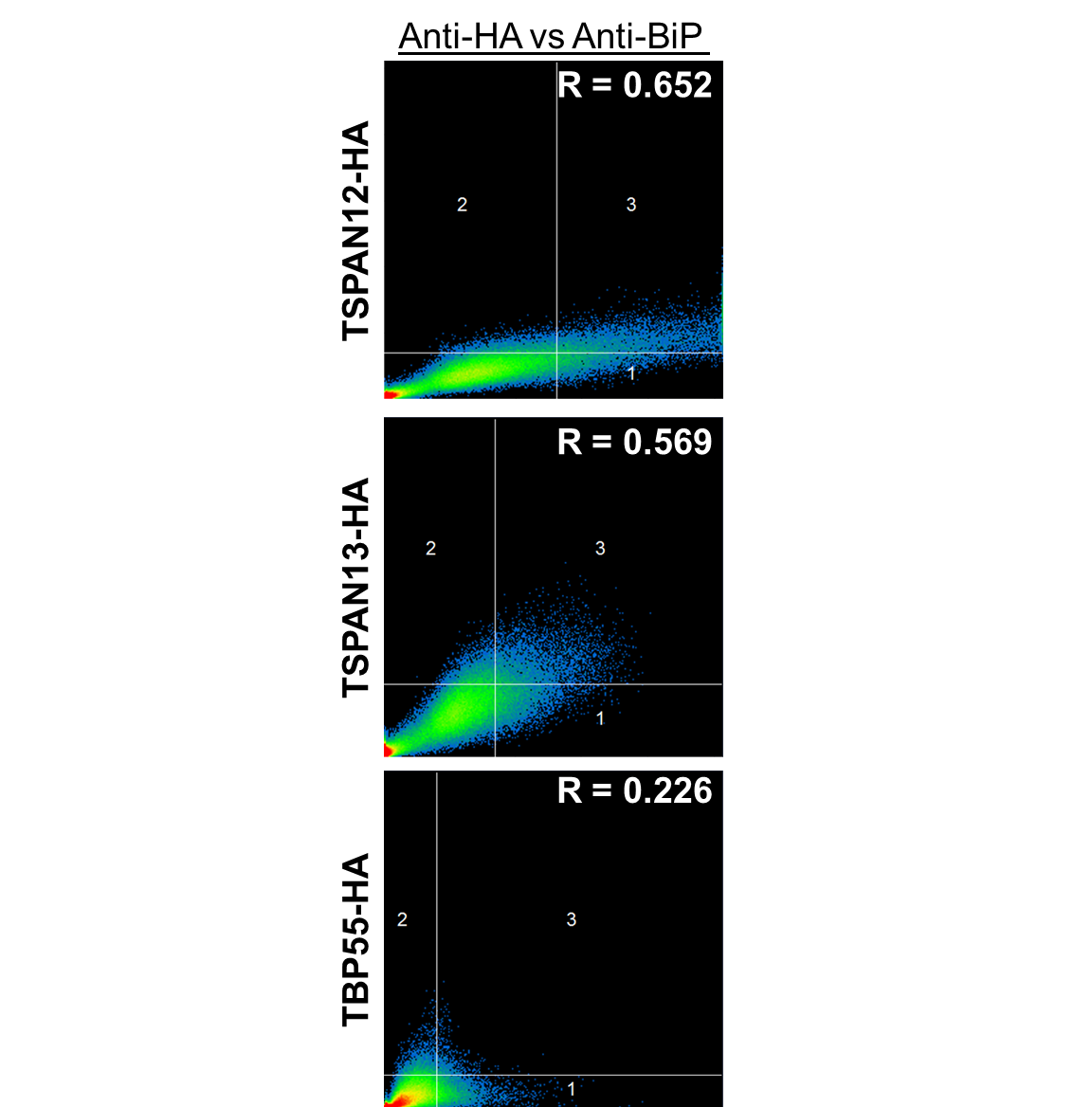

Supplement: S5 Fig — Scatter plots of immunofluorescence images from Fig 3A were generated using the “Colocalization” tool of Zen software (Carl Zeiss, Germany) between anti-HA and anti-BiP in TSPAN12-HA (upper panel) and TSPAN13-HA (middle panel) and TBP55-HA (lower panel) respectively. Colocalization coefficient (R; in parentheses) was computed using the same software. (TIF) [file ppat.1012151.s005.tif]

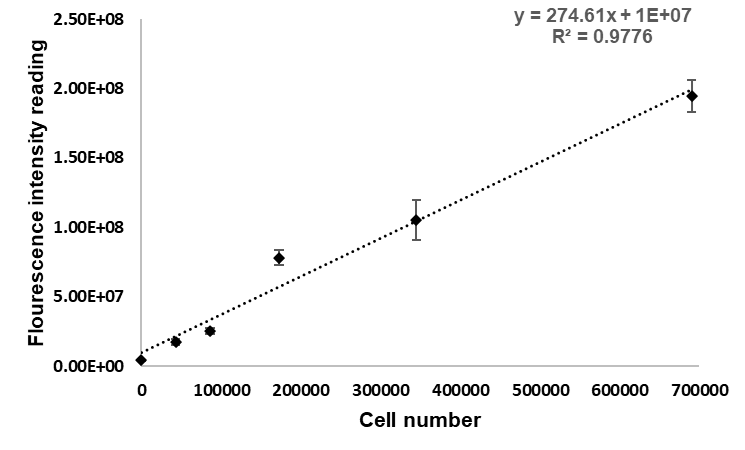

Supplement: S6 Fig — CellTracker Green stained HA-mock transformants were distributed into different wells in collagen-coated 96-well plate in different cell number. Fluorescence intensity was measured after 40 minutes incubation by excitation at 492 nm and emission at 532 nm right after incubation. Triplicate wells were used for each cell number. The linear correlation between cell dye stained trophozoites cell number and fluorescence intensity validate the robustness of the cell adhesion assay. (TIF) [file ppat.1012151.s006.tif]

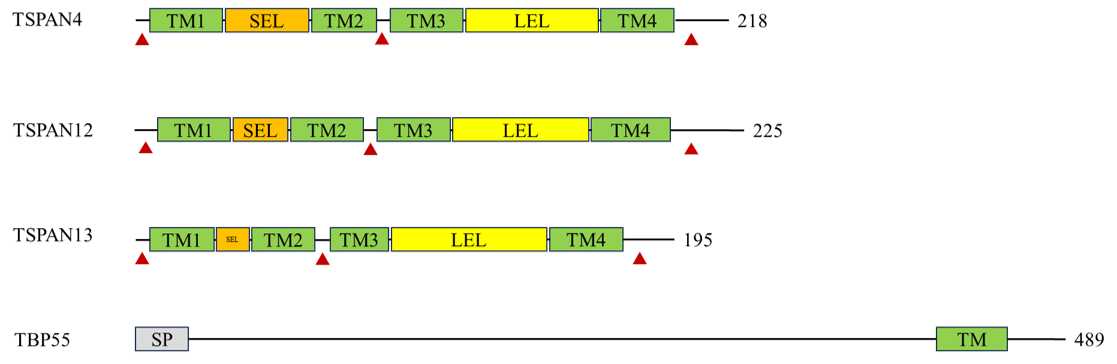

Supplement: S7 Fig — TM (Transmembrane domain), SEL (Small extracellular loop), LEL (Large extracellular loop), SP (Signal peptide), red triangles stand for intracellular domains of tetraspanins. (TIF) [file ppat.1012151.s007.tif]

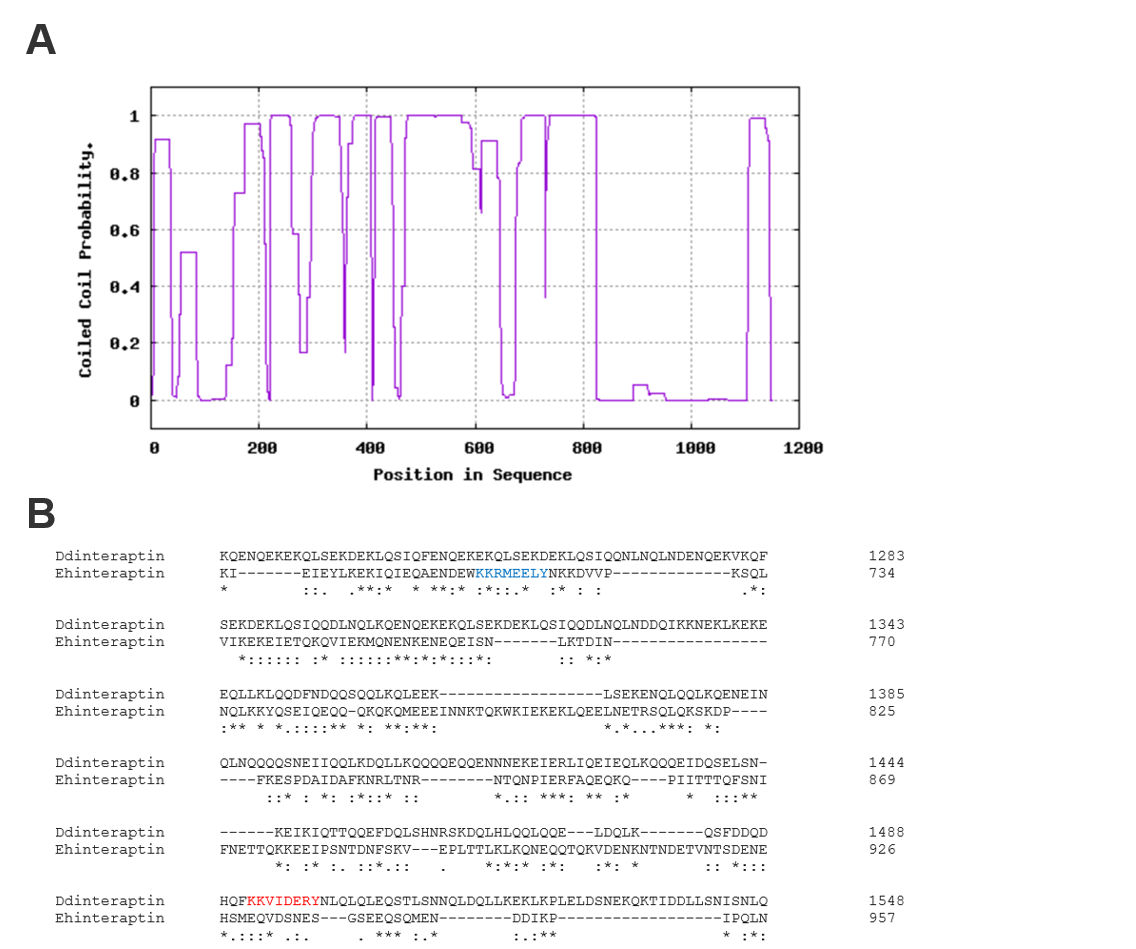

Supplement: S8 Fig — (A) Multiple coiled-coil structures were predicted in Ehinteraptin (EHI_148910) by MARCOIL toolkit (https://toolkit.tuebingen.mpg.de/tools/marcoil) which is a HMM model for coiled-coil domains. (B) Multiple amino acids sequence alignment between Ddinteraptin (AF057019) and Ehinteraptin (EHI_148910) were built by clustal W algorithm. The putative tyrosine phosphorylation site ‘KKVIDERY’ in Ddinteraptin is shown in blue, while the similar motif in Ehinteraptin ‘KKRMEELY’ is shown in red. (TIF) [file ppat.1012151.s008.tif]

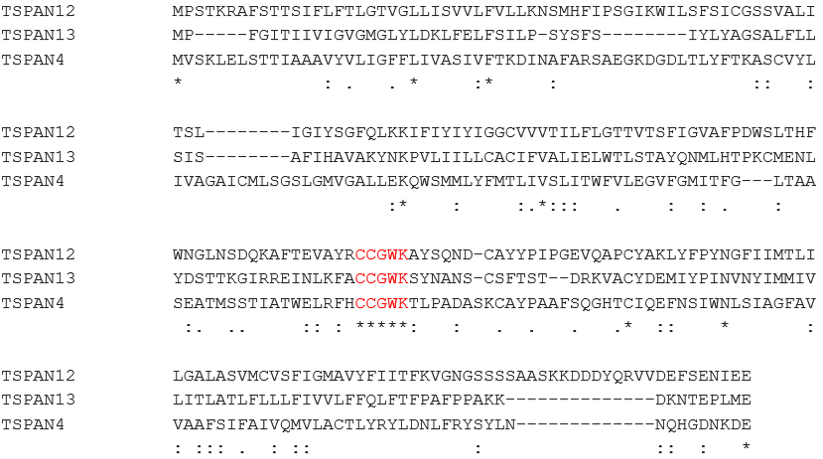

Supplement: S9 Fig — Multiple amino acids sequence alignment of Entamoeba histolytica TSPAN4(EHI_075690), TSPAN12(EHI_091490), TSPAN13(EHI_107790) was established by clustal W algorithm (https://www.genome.jp/tools-bin/clustalw). The highly conserved CCG motifs and the following conserved tryptophan and lysine residues located at large extracellular loops are shown in red font. (TIF) [file ppat.1012151.s009.tif]
