# Supplementary material for: Tetraspanin-enriched microdomains play an important role in pathogenesis in the protozoan parasite Entamoeba histolytica
Source: PLoS Pathog. 2024 Oct 3;20(10):e1012151. doi: 10.1371/journal.ppat.1012151 (PMC11478834; doi:10.1371/journal.ppat.1012151)
Supplement: S1 Table — Experimental details referred to Table 1 description but with the bait protein HA-tagged TSPAN4. The list order is sorted by frequency of identification firstly and mean of quantitative value secondly. (DOCX) [file ppat.1012151.s010.docx]

**S1 Table. Mass-spectrometry results of HA-tagged TSPAN4 in co-immunoprecipitation.** Co-IP assay followed by mass-spectrometry analysis were performed as described in Materials and methods. Frequency of identification indicates the frequency for one protein to be detected in an exclusive or enriched manner in three independent trials. Mean of quantification value suggests the mean of quantitative value (normalized total spectra) calculated by scaffold 5 software, the value outside the parenthesis stands for HA-tagged TSPAN4 sample while the value inside the parenthesis stands for mock control. The order is sorted by frequency of identification firstly, and the mean of quantitative value secondly.

| **Accession number** | **Frequency of identification** | **Mean of quantitative value** | **Molecular weight**  **(kDa)** | **Annotation** |
| --- | --- | --- | --- | --- |
| EHI_001100 | 3 | 105.3 (0.4) | 54.8 | TBP55 |
| EHI_107790 | 3 | 19.0 (0) | 22.2 | TSPAN13 |
| EHI_075690 | 3 | 14.5 (0) | 24.0 | TSPAN4 |
| EHI_091490 | 3 | 13.6 (0) | 24.8 | TSPAN12 |
| EHI_148910 | 3 | 6.8 (0) | 135.2 | *Eh*interaptin |
| EHI_014030 | 2 | 85.1 (34.7) | 117.6 | Proton-translocating NAD(P) (+) transhydrogenase |
| EHI_167300 | 2 | 71.0 (14.8) | 24.3 | Grainin 1 |
| EHI_012270 | 2 | 27.3 (0.8) | 144.3 | Hgl 2 |
| EHI_183510 | 2 | 22.3 (0) | 185.9 | Nup210 |
| EHI_076870 | 2 | 12.5 (3.1) | 33.6 | Steroid 5-alpha reductase |
| EHI_059830 | 2 | 9.1 (2.4) | 99.3 | CPBF8 |
| EHI_152940 | 2 | 8.0 (0) | 124.0 | SMC domain containing protein |
| EHI_015380 | 2 | 7.6 (0.5) | 125.4 | Immuno-dominant variable surface antigen |
| EHI_164430 | 2 | 6.3 (1.1) | 53.9 | Actinin-like protein |
| EHI_163240 | 2 | 6.1 (2.0) | 25.9 | Phosphatidate cytidylyltransferase |
| EHI_048240 | 2 | 5.9 (1.3) | 20.0 | Hypothetical protein |
| EHI_181250 | 2 | 5.6 (2.3) | 21.8 | Rho-related protein RacA |
| EHI_028950 | 2 | 5.3 (0) | 49.5 | Chitobiosyldiphosphodolichol beta-mannosyltransferase |
