# Supplementary material for: Tetraspanin-enriched microdomains play an important role in pathogenesis in the protozoan parasite Entamoeba histolytica
Source: PLoS Pathog. 2024 Oct 3;20(10):e1012151. doi: 10.1371/journal.ppat.1012151 (PMC11478834; doi:10.1371/journal.ppat.1012151)
Supplement: S2 Table — Experimental details referred to Table 1 description but with the bait protein HA-tagged TSPAN12. The list order is sorted by frequency of identification firstly and mean of quantitative value secondly. (DOCX) [file ppat.1012151.s011.docx]

**S2 Table. Mass-spectrometry results of HA-tagged TSPAN12 in co-immunoprecipitation.** Co-IP assay followed by mass-spectrometry analysis were performed as described in Materials and methods. Frequency of identification indicates the frequency for one protein to be detected in an exclusive or enriched manner in three independent trials. Mean of quantification value suggests the mean of quantitative value (normalized total spectra) calculated by scaffold 5 software, the value outside the parenthesis stands for HA-tagged TSPAN12 sample while the value inside the parenthesis stands for mock control. The order is sorted by frequency of identification firstly, and the mean of quantitative value secondly.

| **Accession number** | **Frequency of identification** | **Mean of quantitative value** | **Molecular weight**  **(kDa)** | **Annotation** |
| --- | --- | --- | --- | --- |
| EHI_001100 | 3 | 65.5 (0.1) | 54.8 | TBP55 |
| EHI_101280 | 3 | 29.2 (0.1) | 133.9 | Tyrosine kinase domain containing |
| EHI_091490 | 3 | 15.7 (0) | 24.8 | TSPAN12 |
| EHI_107790 | 3 | 11.3 (0) | 22.2 | TSPAN13 |
| EHI_075690 | 3 | 5.3 (0) | 24.0 | TSPAN4 |
| EHI_086380 | 3 | 4.1 (1.8) | 34.9 | Hypothetical protein |
| EHI_200800 | 3 | 2.1 (0) | 14.9 | Hypothetical protein |
| EHI_004590 | 2 | 29.3 (12.7) | 91.2 | Coatomer subunit beta |
| EHI_088220 | 2 | 19.4 (8.3) | 103.5 | COPB2 COPI coat complex subunit beta 2 |
| EHI_147010 | 2 | 19.3 (5.0) | 77.3 | Long chain fatty acid CoA ligase |
| EHI_040700 | 2 | 18.6 (7.2) | 95.4 | Coatomer subunit gamma |
| EHI_165070 | 2 | 16.6 (4.3) | 36.7 | Short chain dehydrogenase |
| EHI_148910 | 2 | 13.0 (1.1) | 135.2 | *Eh*interaptin |
| EHI_138080 | 2 | 12.3 (2.2) | 23.4 | Tmp21 |
| EHI_197510 | 2 | 10.4 (4.4) | 29.9 | EF-hand calcium-binding domain containing |
| EHI_141860 | 2 | 9.2 (2.2) | 67.8 | Phosphoinositide phosphatase |
| EHI_095870 | 2 | 9.0 (2.0) | 103.9 | Serine-threonine rich protein |
| EHI_131050 | 2 | 8.5 (5.4) | 51.6 | PHD-type domain containing protein |
| EHI_042170 | 2 | 7.7 (3.6) | 56.9 | Aminoacyl-histidine dipeptidase |
| EHI_050800 | 2 | 7.7 (0) | 64.5 | *Eh*CP |
| EHI_015380 | 2 | 7.4 (1.7) | 125.4 | immuno-dominant variable surface antigen |
| EHI_163260 | 2 | 7.3 (3.2) | 28.8 | Copine, putative |
| EHI_124330 | 2 | 6.9 (2.4) | 11.9 | 60S ribosomal protein L36 |
| EHI_076870 | 2 | 6.7 (2.1) | 33.6 | Steroid 5-alpha reductase |
| EHI_107250 | 2 | 6.7 (2.8) | 23.8 | Rab11A |
| EHI_163540 | 2 | 6.6 (2.2) | 36.6 | B-keto acyl reductase |
| EHI_163740 | 2 | 6.1 (0.2) | 68.4 | BOP1 |
| EHI_009860 | 2 | 6.1 (0.4) | 63.0 | Pescadillo homolog |
| EHI_098830 | 2 | 5.6 (1.0) | 161.3 | Nucleoporin |
| EHI_163480 | 2 | 5.6 (2.2) | 81.2 | Hsp90 |
| EHI_118810 | 2 | 5.2 (1.7) | 122.2 | Protein kinase domain containing protein |
| EHI_042870 | 2 | 5.1 (0) | 75.5 | Cell surface protease gp63 |
