# Supplementary material for: Tetraspanin-enriched microdomains play an important role in pathogenesis in the protozoan parasite Entamoeba histolytica
Source: PLoS Pathog. 2024 Oct 3;20(10):e1012151. doi: 10.1371/journal.ppat.1012151 (PMC11478834; doi:10.1371/journal.ppat.1012151)
Supplement: S3 Table — Experimental details referred to Table 1 description but with the bait protein HA-tagged TSPAN13. The list order is sorted by frequency of identification firstly and mean of quantitative value secondly. (DOCX) [file ppat.1012151.s012.docx]

**S3 Table. Mass-spectrometry results of HA-tagged TSPAN13 in co-immunoprecipitation.** Co-IP assay followed by mass-spectrometry analysis were performed as described in Materials and methods. Frequency of identification indicates the frequency for one protein to be detected in an exclusive or enriched manner in three independent trials. Mean of quantification value suggests the mean of quantitative value (normalized total spectra) calculated by scaffold 5 software, the value outside the parenthesis stands for HA-tagged TSPAN13 sample while the value inside the parenthesis stands for mock control. The order is sorted by frequency of identification firstly, and the mean of quantitative value secondly.

| **Accession number** | **Frequency of identification** | **Mean of quantitative value** | **Molecular weight**  **(kDa)** | **Annotation** |
| --- | --- | --- | --- | --- |
| EHI_199590 | 3 | 43.7 (12.9) | 73.5 | Hsp70, Bip |
| EHI_148910 | 3 | 31.1 (1.5) | 135.2 | *Eh*interaptin |
| EHI_183510 | 3 | 29.0 (1.9) | 185.9 | Nup210 |
| EHI_061870 | 3 | 22.4 (0) | 235.2 | Protein kinase domain-containing protein |
| EHI_074180 | 3 | 15.4 (3.4) | 35.1 | Cysteine proteinase 1 |
| EHI_033710 | 3 | 15.2 (3.9) | 34.7 | Cysteine proteinase 2 |
| EHI_152940 | 3 | 15.1 (1.5) | 124.0 | SMC domain containing protein |
| EHI_107790 | 3 | 15.0 (0) | 22.2 | TSPAN13 |
| EHI_095870 | 3 | 13.0 (3.3) | 103.9 | Serine-threonine rich protein |
| EHI_045120 | 3 | 12.4 (2.2) | 88.4 | Cell division cycle protein 48 |
| EHI_166800 | 3 | 12.0 (2.3) | 11.1 | Ubiquitin |
| EHI_010010 | 3 | 9.5 (3.5) | 33.3 | Nup54 |
| EHI_049740 | 3 | 9.1 (0) | 39.8 | Ubiquitin-conjugating enzyme family protein |
| EHI_058330 | 3 | 9.0 (0) | 31.6 | Gal/GalNAc lectin light subunit |
| EHI_068160 | 3 | 8.4 (0) | 241.7 | Transmembrane tyrosine kinase |
| EHI_064500 | 3 | 7.1 (0) | 133.2 | Protein tyrosine kinase domain-containing protein |
| EHI_082590 | 3 | 7.1 (0) | 120.9 | Glutamic acid-rich protein precursor putative |
| EHI_175420 | 3 | 6.5 (0) | 99.6 | Hypothetical protein |
| EHI_110160 | 3 | 6.2 (1.2) | 53.6 | Hypothetical protein |
| EHI_169970 | 3 | 6.2 (2.0) | 42.8 | Hypothetical protein |
| EHI_153160 | 3 | 5.9 (0) | 67.9 | Transmembrane protein |
| EHI_022950 | 3 | 5.5 (0) | 41.4 | DnaJ family protein |
| EHI_100480 | 3 | 5.4 (1.6) | 23.5 | MIR domain protein |
| EHI_021560 | 3 | 5.0 (0) | 43.0 | Thioredoxin, putative |
| EHI_138060 | 3 | 4.5 (0) | 30.5 | SPRY domain-containing protein 7 |
| EHI_037140 | 3 | 4.4 (0.7) | 128.9 | Transmembrane kinase 39 |
| EHI_138330 | 3 | 4.3 (0) | 102.0 | Uncharacterized protein |
| EHI_067510 | 3 | 3.9 (0) | 32.6 | UIp1a |
| EHI_030830 | 3 | 3.8 (0.7) | 113.9 | Calcium-transporting ATPase |
| EHI_055720 | 3 | 3.8 (0) | 65.8 | Nuclear pore protein |
| EHI_142960 | 3 | 3.6 (0.3) | 60.6 | Wntless-like transmembrane domain-containing protein |
| EHI_026310 | 3 | 3.6 (0) | 51.1 | Uncharacterized protein |
| EHI_158120 | 3 | 3.2 (0) | 58.7 | Uncharacterized protein |
| EHI_141990 | 3 | 3.1 (1.3) | 28.6 | 60S ribosomal protein L7, putative |
| EHI_098250 | 3 | 2.9 (0.7) | 33.3 | MutT/nudix family protein |
| EHI_118030 | 3 | 2.9 (0.7) | 44.8 | Ribosome biogenesis protein WDR12 homolog |
| EHI_051730 | 3 | 2.9 (0) | 45.4 | Uridine/cytidine kinase |
| EHI_079720 | 3 | 2.7 (0) | 46.2 | DnaJ family protein |
| EHI_069270 | 3 | 2.7 (0.3) | 57.1 | Transmembrane protein |
| EHI_006730 | 3 | 2.6 (0) | 41.8 | Uncharacterized protein |
| EHI_070680 | 3 | 2.6 (0) | 56.4 | Uncharacterized protein |
| EHI_163740 | 3 | 2.6 (0.2) | 68.4 | Ribosome biogenesis protein BOP1 homolog |
| EHI_011940 | 3 | 2.5 (0) | 82.9 | Dolichyl diphosphooligosaccharide protein glycotransferase |
| EHI_001100 | 3 | 2.4 (0) | 54.9 | TBP55 |
| EHI_118810 | 3 | 2.3 (0.7) | 122.2 | Protein kinase domain containing protein |
| EHI_050800 | 3 | 2.3 (0) | 64.5 | *Eh*CP |
| EHI_175040 | 3 | 1.9 (0.5) | 66.7 | Uncharacterized protein |
| EHI_185250 | 3 | 1.8 (0) | 49.9 | Uncharacterized protein |
| EHI_148170 | 3 | 1.7 (0) | 50.4 | Serine/threonine-protein phosphatase 2A 55 kDa regulatory subunit B |
| EHI_010850 | 3 | 1.7 (0) | 35.0 | *Eh*CP-A7 |
| EHI_076860 | 3 | 1.5 (0) | 45.3 | Uncharacterized protein |
| EHI_179400 | 3 | 1.5 (0) | 39.2 | Nuclear pore protein, putative |
| EHI_200840 | 3 | 1.4 (0) | 54.2 | Signal recognition particle receptor alpha subunit, putative |
| EHI_125710 | 3 | 1.3 (0) | 34.5 | Uncharacterized protein |
| EHI_120600 | 3 | 1.3 (0) | 34.3 | Uncharacterized protein |
| EHI_054180 | 3 | 1.3 (0) | 96.6 | Protein SEY1 homolog 2 |
| EHI_112870 | 3 | 1.2 (0) | 53.7 | 3-ketoacyl-CoA synthase |
