# Supplementary material for: Tetraspanin-enriched microdomains play an important role in pathogenesis in the protozoan parasite Entamoeba histolytica
Source: PLoS Pathog. 2024 Oct 3;20(10):e1012151. doi: 10.1371/journal.ppat.1012151 (PMC11478834; doi:10.1371/journal.ppat.1012151)
Supplement: S4 Table — Experimental details referred to Table 1 description but with the bait protein HA-tagged Ehinteraptin. The Co-IP and MS analysis were conducted twice independently. The list order is sorted by frequency of identification firstly and mean of quantitative value secondly. (DOCX) [file ppat.1012151.s013.docx]

**S4 Table. Mass-spectrometry results of HA-tagged *Eh*interaptin in co-immunoprecipitation.** Co-IP assay followed by mass-spectrometry analysis were performed as described in Materials and methods. Frequency of identification indicates the frequency for one protein to be detected in an exclusive or enriched manner in two independent trials. Mean of quantification value suggests the mean of quantitative value (normalized total spectra) calculated by scaffold 5 software, the value outside the parenthesis stands for HA-tagged *Eh*interaptin sample while the value inside the parenthesis stands for mock control. The order is sorted by frequency of identification firstly, and the mean of quantitative value secondly.

| **Accession number** | **Frequency of identification** | **Mean of quantitative value** | **Molecular weight (kDa)** | **Annotation** |
| --- | --- | --- | --- | --- |
| EHI_110180 | 2 | 192.3 (49.4) | 246.6 | Myosin heavy chain |
| EHI_148910 | 2 | 85.5 (1.9) | 135.2 | *Eh*interaptin |
| EHI_122800 | 2 | 22.6 (2.5) | 186.9 | Coronin |
| EHI_000590 | 2 | 18.9 (0) | 29.8 | 40S ribosomal protein S6 |
| EHI_122740 | 2 | 17.7 (8.2) | 51.5 | Nucleolar protein Nop56, putative |
| EHI_140720 | 2 | 17.3 (0) | 151.5 | Myosin heavy chain |
| EHI_095870 | 2 | 15.1 (0) | 103.9 | Serine-threonine rich protein |
| EHI_118840 | 2 | 13.2 (5.7) | 32.5 | Fibrillarin, putative |
| EHI_021270 | 2 | 11.1 (0) | 169.8 | Villidin, putative |
| EHI_164460 | 2 | 11.1 (0) | 83.2 | Leucine-rich repeat containing protein |
| EHI_115300 | 2 | 6.8 (1.9) | 54.1 | Centromere/microtubule binding protein cbf5, putative |
| EHI_178570 | 2 | 6.7 (0) | 56.8 | Cpn60 |
| EHI_026440 | 2 | 6.2 (1.3) | 28.7 | RNA recognition motif domain containing protein |
| EHI_154330 | 2 | 6.2 (0) | 111.5 | Calponin-homology (CH) domain-containing protein |
| EHI_023500 | 2 | 5.8 (0) | 17.4 | Calmodulin, putative |
| EHI_001950 | 2 | 5.5 (2.5) | 74 | Heat shock protein 70 family |
| EHI_095610 | 2 | 5.2 (0) | 22.8 | 60S ribosomal protein L16-B, putative |
| EHI_177190 | 2 | 5.1 (0) | 84 | Hypothetical protein |
| EHI_152940 | 1 | 56.2 (0) | 124 | SMC domian containing protein |
