# Supplementary material for: Tetraspanin-enriched microdomains play an important role in pathogenesis in the protozoan parasite Entamoeba histolytica
Source: PLoS Pathog. 2024 Oct 3;20(10):e1012151. doi: 10.1371/journal.ppat.1012151 (PMC11478834; doi:10.1371/journal.ppat.1012151)
Supplement: S5 Table — EhCPBFs and EhCPs proteins found in TSPANs-HA and TBP55-HA proteome. The upper panel of TSPAN4-HA, TSPAN12-HA, TSPAN13-HA, TBP55-HA indicate the number of independent trials the protein was pulled down by the certain bait protein. The lower panel suggests the mean QV that the protein detected in TSPANs or TBP55, the number inside bracket suggests mean QV that the protein detected in the mock control. ND, no detection. (DOCX) [file ppat.1012151.s014.docx]

**S5 Table. *Eh*CPBFs and *Eh*CPs proteins pulled-down by amebic TSPANs and their binding proteins.** *Eh*CPBFs and *Eh*CPs proteins found in TSPANs-HA, TBP55-HA and *Eh*interaptin-HA proteome. The upper panel of TSPAN4-HA, TSPAN12-HA, TSPAN13-HA, TBP55-HA and *Eh*interaptin-HA indicate the number of independent trials the protein was pulled down by the certain bait protein. The lower panel suggests the mean QV that the protein detected in corresponding proteome, the number inside bracket suggests mean QV that the protein detected in the mock control. ND, no detection.

| **Accession number** | **Annotation** | **TSPAN4-HA** | **TSPAN12-HA** | **TSPAN13-HA** | **TBP55-HA** | ***Eh*interaptin-HA** |
| --- | --- | --- | --- | --- | --- | --- |
| EHI_164800 | CPBF1 | ND | 1 out of 3  3.3 (1.0) | 2 out of 3  3.8 (0.8) | ND | ND |
| EHI_161650 | CPBF3 | ND | 1 out of 3  3.1 (1.5) | 1 out of 3  5.2 (0.9) | ND | ND |
| EHI_012340 | CPBF4 | 1 out of 3  1.5 (0) | ND | 1 out of 3  1.6 (0.5) | ND | 1 out of 2  3.3 (0) |
| EHI_178470 | CPBF6 | ND | ND | 1 out of 3  0.4 (0) | ND | ND |
| EHI_059830 | CPBF8 | 2 out of 3  9.1 (0) | 1 out of 3  5.4 (1.0) | 2 out of 3  2.2 (0.5) | ND | ND |
| EHI_074180 | *Eh*CP-A1 | 2 out of 2  8.0 (0) | 2 out of 3  9.1 (2.4) | 3 out of 3  15.4 (3.4) | ND | ND |
| EHI_033710 | *Eh*CP-A2 | ND | 2 out of 3  6.3 (2.6) | 3 out of 3  15.2 (3.9) | 1 out of 3  16.9 (5.8) | ND |
| EHI_168240 | *Eh*CP-A5 | ND | ND | 2 out of 3  6.7 (1.1) | ND | ND |
| EHI_010850 | *Eh*CP-A7 | ND | ND | 3 out of 3  1.7 (0) | ND | ND |
| EHI_050800 | *Eh*CP | 2 out of 3  2.1 (0) | 2 out of 3  7.7 (0) | 3 out of 3  2.3 (0) | 1 out of 3  2.1 (0) | ND |
