# Supplementary material for: Tetraspanin-enriched microdomains play an important role in pathogenesis in the protozoan parasite Entamoeba histolytica
Source: PLoS Pathog. 2024 Oct 3;20(10):e1012151. doi: 10.1371/journal.ppat.1012151 (PMC11478834; doi:10.1371/journal.ppat.1012151)
Supplement: S6 Table — The alignment was conducted by ClustalW multiple sequence alignment toolkit. (DOCX) [file ppat.1012151.s015.docx]

**S6 Table. Percentage of amino acid identity among TSPAN4, TSPAN12 and TSPAN13.** The alignment was conducted by ClustalW multiple sequence alignment toolkit.

| **Identity%** | **TSPAN4** | **TSPAN12** | **TSPAN13** |
| --- | --- | --- | --- |
| TSPAN4 | 100.0 | 13.8 | 11.8 |
| TSPAN12 |  | 100.0 | 17.9 |
| TSPAN13 |  |  | 100.0 |
